# Evolution of DNMT2 in drosophilids: Evidence for positive and purifying selection and insights into new protein (pathways) interactions

**DOI:** 10.1590/1678-4685-GMB-2017-0056

**Published:** 2018-03-26

**Authors:** Gilberto Cavalheiro Vieira, Marícia Fantinel D’Ávila, Rebeca Zanini, Maríndia Deprá, Vera Lúcia da Silva Valente

**Affiliations:** 1 Departamento de Genética, Instituto de Biociências, Universidade Federal do Rio Grande do Sul (UFRGS), Porto Alegre, RS, Brazil; 2 Departamento de Zoologia e Ciências Biológicas, Universidade Federal de Santa Maria (UFSM), Palmeira das Missões, RS, Brazil; 3 Programa de Pós-Graduação em Genética e Biologia Molecular, Universidade Federal do Rio Grande do Sul (UFRGS), Porto Alegre, RS, Brazil; 4 Programa de Pós-Graduação em Biologia Animal, Universidade Federal do Rio Grande do Sul (UFRGS), Porto Alegre, RS, Brazil

**Keywords:** Drosophila, Dnmt2, positive selection, epigenetic, positive-destabilizing selection

## Abstract

The DNA methyltransferase 2 (DNMT2) protein is the most conserved member of the DNA methyltransferase family. Nevertheless, its substrate specificity is still controversial and elusive. The genomic role and determinants of DNA methylation are poorly understood in invertebrates, and several mechanisms and associations are suggested. In *Drosophila*, the only known DNMT gene is *Dnmt2*. Here we present our findings from a wide search for *Dnmt2* homologs in 68 species of Drosophilidae. We investigated its molecular evolution, and in our phylogenetic analyses the main clades of Drosophilidae species were recovered. We tested whether the *Dnmt2* has evolved neutrally or under positive selection along the subgenera *Drosophila* and *Sophophora* and investigated positive selection in relation to several physicochemical properties. Despite of a major selective constraint on *Dnmt2*, we detected six sites under positive selection. Regarding the DNMT2 protein, 12 sites under positive-destabilizing selection were found, which suggests a selection that favors structural and functional shifts in the protein. The search for new potential protein partners with DNMT2 revealed 15 proteins with high evolutionary rate covariation (ERC), indicating a plurality of DNMT2 functions in different pathways. These events might represent signs of molecular adaptation, with molecular peculiarities arising from the diversity of evolutionary histories experienced by drosophilids.

## Introduction

Methylation of cytosine to form 5-methylcytosine is one of the most important epigenetic marks acting in the control of gene expression without altering the DNA nucleotide sequence. Cytosine methylation plays a critical role in the regulation of gene expression in higher eukaryotes. It is established by DNA methyltransferases (DNMTs), classified into three subfamilies: DNMT1, DNMT2 e DNMT3. The smallest eukaryotic methyltransferase, DNMT2, is most widely distributed in animals, fungi, protists, and plants (Ponger and Li, 2005). Dnmt2 was first identified in mice and humans and appears to be well conserved among eukaryotes (Okano *et al.*, 1998; Yoder and Bestor, 1998). This enzyme is the only DNMT found in dipterans, including *Drosophila* (organisms “Dnmt2-only”) (Kucharski *et al.*, 2008). In agreement with this structural conservation, different methods in various organisms have shown DNMT2 to have DNA methyltransferase activity (Hermann *et al.*, 2003; Tang *et al.*, 2003; Fisher *et al.*, 2004; Katoh *et al.*, 2006). However, according to analyses of human and *Entamoeba* enzymes, its catalytic activity on DNA is very weak (Hermann *et al.*, 2003; Fisher *et al.*, 2004). In addition, cytosine methylation of non-coding RNA (ncRNA) plays an important role in the epigenetic landscape. The functions of some tRNA modifications remain obscure. However, Tuorto *et al.* (2012) have shown that cytosine-C5 methylation of tRNAs is associated with their structural stability and the rates of protein synthesis in mammals. Cytosine-C5 methylation is involved in protecting tRNA against degradation induced by cellular stress events (Saikia *et al.*, 2012).

Furthermore, studies have associated DNMT2 with RNA interference in *Dictyostelium* (Kuhlmann *et al.*, 2005) and covalent histone modification in *Drosophila* (Kunert *et al.*, 2003), suggesting a role of DNMT2 in epigenetic regulation. Several studies have shown the occurrence of DNA methylation phenomena in *Drosophila* species (Lyko *et al.*, 2000; Kunert *et al.*, 2003; Marhold *et al.*, 2004). However, recently, Takayama *et al.* (2014) showed that methylation in the genome of *Drosophila melanogaster* probably is independent of DNMT2 activity. These findings brought new questions about the epigenetic mechanisms involved in the methylation process in evolutionarily related species of drosophilids.

Garcia *et al.* (2007) compared the DNMT2 protein sequences of *D. willistoni* and *D. melanogaster* and found higher conservation at the domains putatively responsible for methyl transfer catalysis and variability in the region containing the specific target recognition domain (TRD). These findings may be indicative of variation in DNMT2 function among organisms, suggesting that the targets – or modularity - of methylation can also vary among species of the same genus. Furthermore, Garcia *et al.* (2007) described sex-specific methylation patterns in *D. willistoni*, not present in *D. melanogaster*. Using the Methylation-Sensitive Restriction Endonuclease (MSRE) technique and Southern blot analysis with specific probes, the results suggested that selection for different targets of methylation might occur between different, but closely related species (Garcia *et al.*, 2007). Furthermore, D’Ávila *et al.* (2010) found phylogenetic correlations in the sex-specific methylation patterns in the species of the *willistoni* subgroup, where *D. willistoni, D. tropicalis* and *D. insularis* (closer related species) shared methylation patterns in ribosomal genes, whereas *D. equinoxialis* and *D. paulistorum* patterns apparently are not restricted to rDNA.

The presence of DNMT2 enzymes in Diptera was described by Marhold *et al.* (2004), revealing that DNMT2 protein sequences are highly conserved in *D. virilis, D. hydei, D. simulans, D. melanogaster* and *D. pseudoobscura*, primarily within the catalytic DNA methyltransferase motifs. The Drosophilidae family is among the most diverse of the Diptera, encompassing more than 4,200 species (Bächli, 2016). Species of this family, especially of the genus *Drosophila*, are widely used in many areas of contemporary biological research. However, only few have been investigated with respect to the occurrence of DNA methylation and the presence of the *Dnmt2* gene. Thus, the present study objectives are: (i) improve the previous search for *Dnmt2* (Marhold *et al.*, 2004; Garcia *et al.*, 2007), including a large number of *Drosophila* species and other Drosophilidae, to evaluate the conservation, or not, of *Dnmt2* in the genus; (ii) test whether the gene and protein are evolving under relaxed selective constraint or positive selection; and (iii), given the current controversial and enigmatic scenario involving the role of DNMT2 among epigenetic mechanisms of drosophilids, an attempt to find potential protein interaction partners of DNMT2 by database searching for protein-protein interactions and via evolutionary rate covariation (ERC) analysis.

Our results indicate, as expected, substantial conservation of DNMT2 catalytic motifs. Nevertheless, the TRD and the connecting region of the two main domains (catalytic and TRD) show some variability among the species examined, including closely related species. We also detected that several sites are under positive selection. These are located in potential regions of protein-protein interaction. The multiplicity of proteins with high ERC values found in the present work supports the hypothesis of DNMT2 can be involved in several networks, through control of gene expression, genomic stability, and in response to stressor events in “Dnmt2-only” organisms, like drosophilids.

## Material and Methods

### Fly stocks

The conservation of DNMT2 in the family Drosophilidae was analyzed in 68 species of *Drosophila*, along with *Zaprionus indianus*, *Z. tuberculatus, Scaptodrosophila latifasciaeformis* and *S. lebanonensis* (Table S1). Most strains were maintained in the laboratory by mass crosses and reared in corn flour culture medium (Marques *et al.*, 1966) in a controlled environment chamber (17 ± 1 °C, 60% r.h.), except for those species for which sequences were obtained directly from GenBank.

### PCR, cloning and sequencing

Genomic DNA was extracted from adult flies following Sassi *et al.* (2005). Primers initially used are described in Marhold *et al.* (2004) (5’ *D. melanogaster* Dnmt2-F: 5’ GTGGCATTGGCGGCATGCATTATGCCT 3’ and *D. melanogaster* Dnmt2-R: 5’ CGATACTTTTGTCGATTCGTTGTTTCTGGC 3’). This pair of primers was designed directed against conserved catalytic motifs of *D. melanogaster* and used to amplify *Dnmt2* sequences from *D. simulans*, *D. hydei* and *D. virilis* (Marhold *et al.*, 2004). In this work, specific primers were designed to *D. willistoni Dnmt2* genes (wDnmt2A-F: 5’ TCACCCACAACCTTGACATT 3’ and wDnmt2C-R: 5’ ACCTTCTCGCAGACACCAA 3’). Both pairs of primers align in similar regions of the gene *Dnmt2*. PCRs were performed in 25 μL volumes containing 20 ng of genomic DNA, 1 U of *Taq* DNA polymerase (Invitrogen, Carlsberg, CA, USA), 1X reaction buffer, 200 μM of each nucleotide, 20 pmol of each primer and 1.5 mM MgCl_2_. The amplification sets consisted of a denaturation step of 95 °C for 5 min, followed by 30 cycles at 95 °C for 40 s, 55 °C to 60 °C for 40 s and 72 °C for 1 min, and then a final extension cycle at 72 °C for 5 min. *Dnmt2* amplicons were directly purified by incubation at 37 ºC for 30 min with Exonuclease I and Shrimp Alkaline Phosphatase (SAP) (both from USB, Cleveland, OH, USA) followed by a 15 min inactivation step at 80 ºC. For the *Dnmt2* amplicons of *D. teissieri, D. ornatifrons, D. ornatipennis* and *D. tropicalis*, fragments were excised of the agarose gel and purified using Illustra GFX PCR DNA kit (GE Healthcare, Pittsburgh, PA, USA). The purified fragments were cloned into pCR4-TOPO plasmids (Invitrogen). DNA sequencing was performed by Macrogen Inc. (South Korea) using the appropriate primers (forward and reverse). The sequences generated by PCR and sequencing were assembled using the GAP 4 software of the Staden Package (Staden, 1996) (Table S2).

### Dot blot analyses

For Dot blot hybridizations, samples of denatured DNA (1 μg) were transferred onto a nylon membrane (Hybond-N+; GE Healthcare Biosciences). The AlkPhos Direct Labelling and Detection System and the CDP-Star kit (GE Healthcare) were used to label and detect nucleic acids according to the manufacturer’s instructions. The *Dnmt2* amplicon from *D. melanogaster* was used as a probe at the stringency temperature of 55 ºC.

### Data collection from public databases

*In silico* searches were performed to identify the complete sequence homologs of *Dnmt2* among 24 sequenced *Drosophila* genomes available in the FlyBase database, using the *Dnmt2* of *D. melanogaster* (Accession number: AAF53163.2) as query (Table S2). The *D. buzzattii* and *D. suzukii Dnmt2* genes were obtained from the *Drosophila buzzatii* Genome Project server and Spotted Wing Fly Base, a dedicated online resource for *D. suzukii* genomics (Table S2).

### Evolutionary analysis

All sequences were aligned using the Muscle tool (Edgar, 2004). The evolutionary relationships among the *Dnmt2* sequences were estimated using Bayesian analysis, which is implemented in MrBayes (Ronquist and Huelsenbeck, 2003), with the evaluation of at least 1,000,000 generations and a burn-in region of 2,500 trees. Each nucleotide sequence was individually translated into its corresponding proteins and aligned using the Muscle tool with default parameter values. For the evolutionary analysis of the amino acid sequences, the Jones-Taylor-Thornton (JTT+G) model was used, as suggested by ProtTest 2.2 (Abascal *et al.*, 2005), in accordance with the Akaike information criterion (Akaike, 1974). A Bayesian analysis of the nucleotide sequences was performed with the general time reversible (GTR) model using the ratio of invariable sites (I) and the gamma distribution of the variable sites (G) model, as suggested in MrModel Test 2.3 (Nylander, 2004). The sequence of *Spodoptera frugiperda* was used as outgroup. For the nucleotide and amino acid divergence analyses, sequences were clustered within species groups to perform a p-distance analysis using the MEGA 7 package (Kumar *et al.*, 2016).

### Analysis of positive selection

To investigate probable selective pressures that shaped the evolution of drosophilid *Dnmt2* genes, we performed a relaxed branch-site test and a strict branch-site test (Yang and Nielsen, 2002; Zhang *et al.*, 2005) using the software CODEML (Yang, 2007) - PAML package. This software tests for positive selection by comparing a series of alternative hypothesis that differ in how variable dN/dS ratio can change in different branches and codons, in which dN/dS > 1 would indicate positive selection and dN/dS < 1 would indicate a purifying selection, due to a selective constraint at the codon level.

The complete sequences of *Dnmt2* genes were used, obtained from *in silico* search, as previously described. The initial maximum likelihood (ML) phylogenetic trees were constructed using the 24 complete sequences (Table S1) by the software PHYML 3.1 (Guindon and Gascuel, 2003). The analysis of the nucleotide sequences was performed with the general time reversible (GTR) model using the ratio of invariable sites (I) and the gamma distribution of the variable sites (G) model, as suggested by MrModel Test 2.3 software (Nylander, 2004).

With the relaxed branch-site test or strict branch-site test, phylogenetic trees are separated into *foreground* branches, at which positive selection is tested, and *background* branches, represented by the other lineages. Both tests use the alternative model (MA), in which the codons in *foreground* are allowed to have a dN/dS > 1, and the *background* codons, dN/dS ≤ 1. The relaxed branch-site test null model (M1a) assumes that evolutionary rates are the same for all sites and branches, with all sites varying dN/dS from 0 and 1. In the null model (restricted MA), the dN/dS > 1 category is fixed to 1, so all sites with dN/dS > 1 are forced to evolve neutrally (dN/dS = 1). We used a log likelihood ratio test (LRT) to infer the positive selection when these values result in a significant value. The significance of the LTR was verified by a χ_2_^2^ null distribution, with critical values of 2.71 for 5% and 5.41 for 1% significance levels, respectively, originated from a null distribution composed of a 50:50 mixture of point mass 0 and χ_1_^2^ (Zhang *et al.*, 2005).

Additionally, we investigated positive selection with respect to several physicochemical properties of the datasets. The MM01 method of McClellan *et al.* (2005) evaluates whether nonsynonymous substitutions favored changes in protein, either structural or functional. The analyses were carried out by *TreeSAAP* 3.2 (McClellan and McCracken, 2001; Woolley *et al.*, 2003; McClellan *et al.*, 2005). First, global deviation from neutrality is verified by a goodness-of-fit test, in which a comparison of neutral expected distribution and observed distribution of the selected physicochemical properties is made. Positive selection is detected in *TreeSAAP* software when the number of inferred amino acid replacements significantly exceeds the number of expected replacements caused by chance alone, given positive z-scores. Stabilizing-selection can be visualized when the magnitude of chance is low (categories 1, 2 and 3), meaning it is a conservative process, while positive-destabilizing selection is represented as a high magnitude of change (categories 6, 7 and 8) (McClellan *et al.*, 2005). Stabilizing selection is defined by McClellan *et al.* (2005) as a selection that tends to maintain the original biochemical attributes of the protein, and positive-destabilizing selection as a selection that favors structural and functional shifts in a region of a protein. In other words, positive-destabilizing selection represents signs of molecular adaptation. To verify which regions were under positive selection (stabilizing and destabilizing) we performed a sliding window analysis using the amino acid properties significant for this type of change (McClellan *et al.*, 2005).

### Potential protein-protein interaction partners

Using the database STRING (Szklarczyk *et al.*, 2015) we conducted a search for predicted protein-protein interactions with DNMT2. To perform the search, the *D. melanogaster* DNMT2 sequence was used as query and data were collected from *D. ananassae*, *D. grimshawi*, *D. pseudoobscura*, *D. virilis*, *D. willistoni* and *D. yakuba*. We also attempted to evaluate the predicted protein-protein partners from the STRING search and find protein partners with DNMT2 by evolutionary rate covariation (ERC) using the ERC Analysis Webserver (Clark *et al.*, 2012, 2013, Findlay *et al.*, 2014).

## Results

### Detection of *Dnmt2* sequences in Drosophilidae species

In a preliminary screen of the presence of *Dnmt2* sequence homologs within the *Drosophila* genomes, we tested 56 species by dot blot analysis (Table S1 and Figure S1). Of these, 54 showed a positive signal for the *Dnmt2* probe (amplicon from *D. melanogaster*), two of which had a weak signal (*D. orena* and *D. polymorpha*). In this assay we observed a strong hybridization signal, primarily in the *melanogaster* group. Nevertheless, hybridization was also detected in the other species, indicating that the *Dnmt2* gene has related sequences in all species groups analyzed.

To verify this homology, we also performed a *Dnmt2* homolog search by PCR amplification in a large number of Drosophilidae species from different *Drosophila* groups (Table S1). Altogether, 61 species were tested by PCR for presence of a *Dnmt2* gene. Thirty species tested positive for *Dnmt2* by PCR. We achieved 20 amplicons for several species of the *Drosophila* genus, including the *Drosophila* subgenus (*guarani, guaramunu, tripunctata, calloptera, immigrans, mesophragmatica, flavopilosa* and *repleta* groups) and the *Sophophora* subgenus (*melanogaster* and *willistoni* groups). The representative species of the *Dorsilopha* subgenus did not show *Dnmt2* amplification with the primers used. The annealing regions of the primers correspond to motif I (forward primer) and motif X (reverse primer) of *Dnmt2*, both belonging to the catalytic domain of the enzyme (Figure S2). The length of the amplified fragments was around 800 bp.

### DNMT2 conservation

The availability of several species maintained in culture chambers in our laboratory and genomic sequences in gene banks allowed us to search for open reading frames encoding DNMT2 in several genomes. For the analyses of gene conservation, all cloned sequences (obtained by direct PCR or cloned) (Table S3) and those obtained by *in silico* search were used in the phylogenetic analysis to investigate the evolutionary pattern and conservation of *Dnmt2* among Drosophilidae species. The sequences were aligned to build a phylogenetic matrix from the 44 analyzed species (Figure S2).

To perform the analyses of *Dnmt2* nucleotide and amino acid divergence, pairwise comparisons of the sequences were conducted (with species clustered by taxonomic group). The highest nucleotide divergence found was between the *willistoni* and *tripunctata* groups, with a p-distance value of 34.47%; whereas the lowest divergence (p-distance value of 10.70%) was detected between the *guarani* and *calloptera* groups (represented only by *D. ornatipennis*) (Table 1, standard error presented at Table S4). The comparison of all nucleotide sequences between species showed that the sequences of *D. willistoni* and *D. suzukii* are more divergent than any other, with a p-distance value of 35.3% (data not shown). When we estimated the average divergence of amino acid sequence pairs within subgenus *Drosophila* and *Sophophora*, it revealed the almost the same internal divergence: 17,8% (S.E. 1.65) and 18.7% (S.E. 1.62), respectively. Pairwise alignment of the amino acid sequences between groups (Table 1) revealed that *willistoni* and *mesophragmatica* groups were more divergent (p-distance value of 31.84%).

**Table 1:** Estimates of evolutionary divergence between sequence pairs of different Drosophilidae species groups. The numbers of amino acid differences per site from the average over all sequence pairs between groups are given below the diagonal. The measures of nucleotide evolutionary divergence are provided above the diagonal. The p-distances are given in percentages.

Groups		1	2	3	4	5	6	7	8	9	10	11	12	13
1	*calloptera*		25.31	26.39	12.89	10.70	26.08	30.53	23.66	29.58	23.87	15.66	22.22	33.33
2	*flavopilosa*	23.32		23.30	24.23	25.77	26.77	30.83	15.12	29.32	16.62	25.62	16.20	32.18
3	*grimshawi*	24.22	18.83		25.31	26.13	26.23	28.62	22.79	27.11	23.15	26.39	20.37	33.95
4	*guaramunu*	10.09	21.08	23.54		15.15	25.23	29.27	22.97	26.72	23.46	13.87	21.14	34.07
5	*guarani*	7.92	24.81	23.92	11.29		25.36	30.54	23.56	29.15	24.74	17.16	22.89	32.95
6	*immigrans*	17.94	20.63	21.08	17.60	18.76		29.89	25.69	28.50	27.93	25.31	25.08	30.83
7	*melanogaster*	25.16	25.41	24.22	24.63	25.95	23.56		29.87	23.72	29.78	29.93	29.24	32.72
8	*mesophragmatica*	20.78	15.70	19.13	18.68	21.72	19.58	25.29		28.46	15.35	24.34	15.64	33.64
9	*obscura*	25.56	26.01	22.57	24.29	24.81	23.02	19.11	25.26		28.91	28.10	26.23	32.61
10	*repleta*	21.82	14.95	18.09	19.88	22.77	20.40	25.15	11.61	25.66		25.31	18.57	33.02
11	*tripunctata*	12.00	23.09	23.99	10.48	12.86	18.89	25.26	20.67	24.96	21.56		22.69	34.47
12	*virilis*	18.39	11.66	15.25	15.92	18.98	15.70	22.22	10.31	21.52	12.41	17.94		30.56
13	*willistoni*	31.17	31.17	28.48	30.72	31.76	27.91	29.55	31.84	27.20	30.19	31.22	28.92	

Standard error values are shown in Table S5.

**Figure 1:**
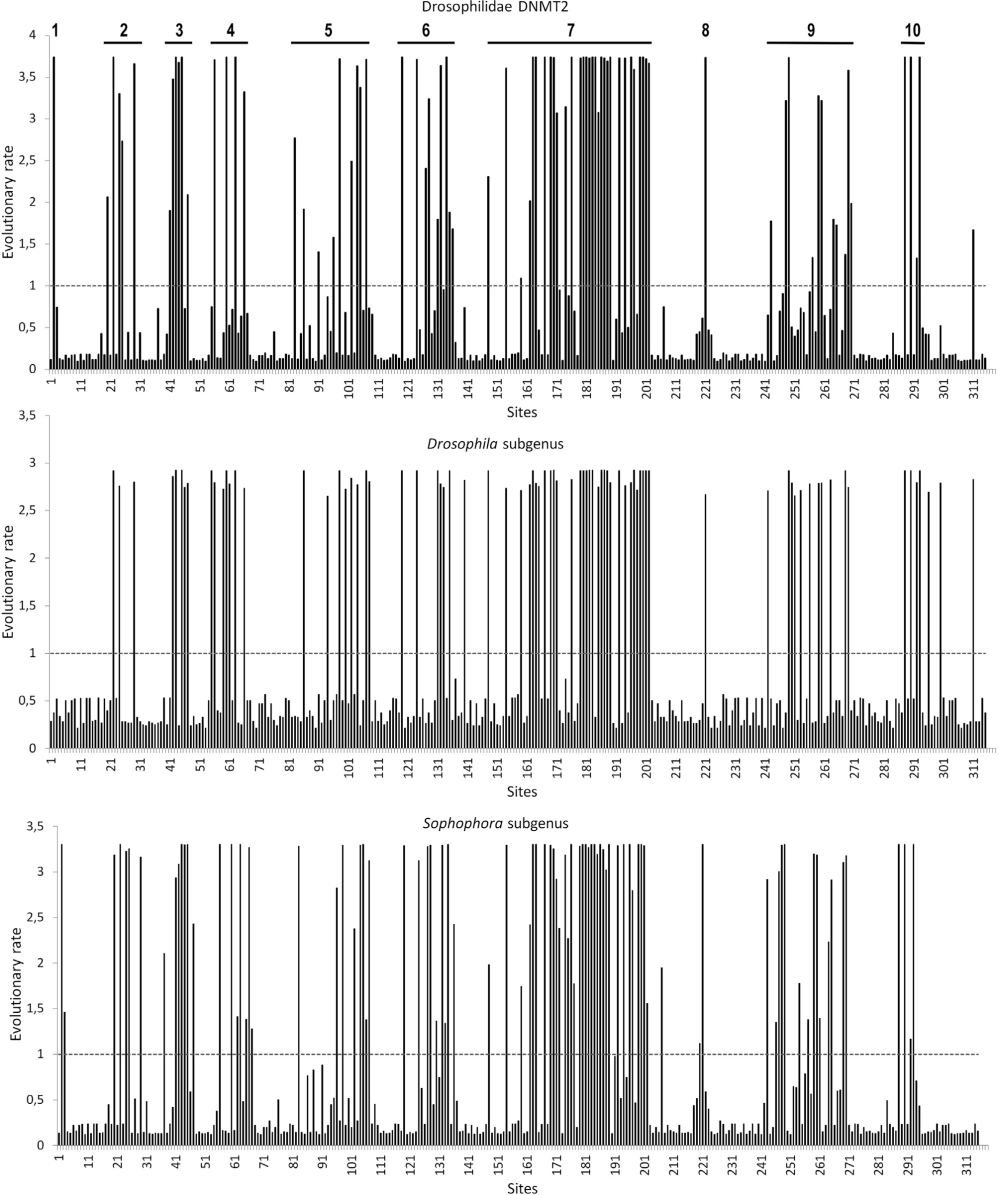
Evolutionary rate from all Drosophilidae DNMT2 complete sequences, *Sophophora* and *Drosophila* subgenus separately. The rates are scaled such that the average evolutionary rate across all sites is 1. All positions containing gaps and missing data are eliminated.

We used the MEGA7 package to compute the mean evolutionary rates at each DNMT2 site. Figure 1 shows the plots, which evolutionary rate are shown to each site for all Drosophilidae complete sequences and for each subgenus separately. Several DNMT2 sites are under evolutionary restrictions, and these sites are mainly part of the catalytic motifs of the enzyme. Nevertheless, 10 regions with sites having high evolutionary rates distributed throughout DNMT2 are evident (Figure 1). This means that sites showing a rate < 1 are evolving slower than average and those with a rate > 1 are evolving faster than average evolutionary rates. Evolutionary rates were estimated under the Jones-Taylor-Thornton (Jones *et al.*, 1992) model (+G).

**Figure 2:**
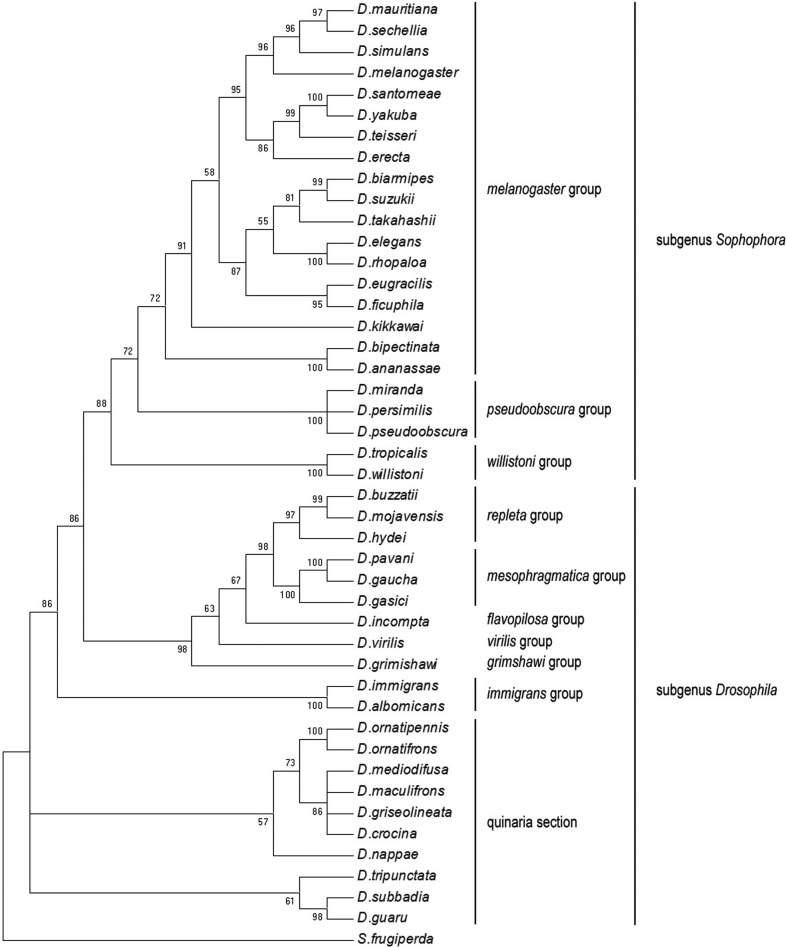
Bayesian phylogenetic inference of the *Dnmt2* gene in Drosophilidae species based on amino acid sequences alignment. Tree generated using the JTT model with a gamma distribution. Sequence of *Spodoptera frugiperda* was used as outgroup.

The phylogeny obtained by amino acid analysis recovered the evolutionary relationship between the subgenera *Drosophila* and *Sophophora* (Figure 2). The *quinaria* section (*Drosophila* subgenus) composed of *guarani, guaramunu, calloptera* and *tripunctata* species groups appears as basal radiation. A second cluster in *Drosophila* subgenus includes the *virilis, repleta, flavopilosa* and *mesophragmatica* species groups (*virilis-repleta* radiation). The *Sophophora* subgenus is composed of the *melanogaster, willistoni* and *obscura* groups, where the *willistoni* group appears as basal to the *obscura-melanogaster* radiation. *D. ananassae* and *D. bipectinata* appear more externally positioned within the *melanogaster* group, likely reflecting the ancestral condition of the *ananassae* subgroup (to which both species belong) within this group (Kopp, 2006; Clark *et al.*, 2007). This placement is confirmed by the correct positioning of the remaining species of the *melanogaster* group, belonging to the *melanogaster*, *takahashii*, *rhopaloa* and *elegans* subgroups, consistent with previous studies (Lewis *et al.*, 2005; Kopp, 2006).

The nucleotide Bayesian inference tree of *Dnmt2* (Figure S3) showed congruence with the tree previously obtained for the Drosophilidae, which was based on nuclear genes and produced using similar methods (Gailey *et al.*, 2000; Tarrio *et al.*, 2001; Da Lage *et al.*, 2007). However, *D. immigrans* and *D. albomicans* sequences were incorrectly positioned, grouping with the *Sophophora* subgenus, but with low statistical support. Also, the *quinaria* section presented polytomy. There is no strong evidence to sustain this finding, and the data from amino acids analysis confirm the correct positioning of this species, grouped with the rest of the *Drosophila* subgenus species, according to previous studies (Throckmorton, 1975; Russo *et al.*, 1995; Kwiatowski and Ayala, 1999), however, again some clades showed low statistical support and the *quinaria* section species grouped with polytomy.

### Long-term evolutionary analysis in drosophilids *Dnmt2*

**Figure 3:**
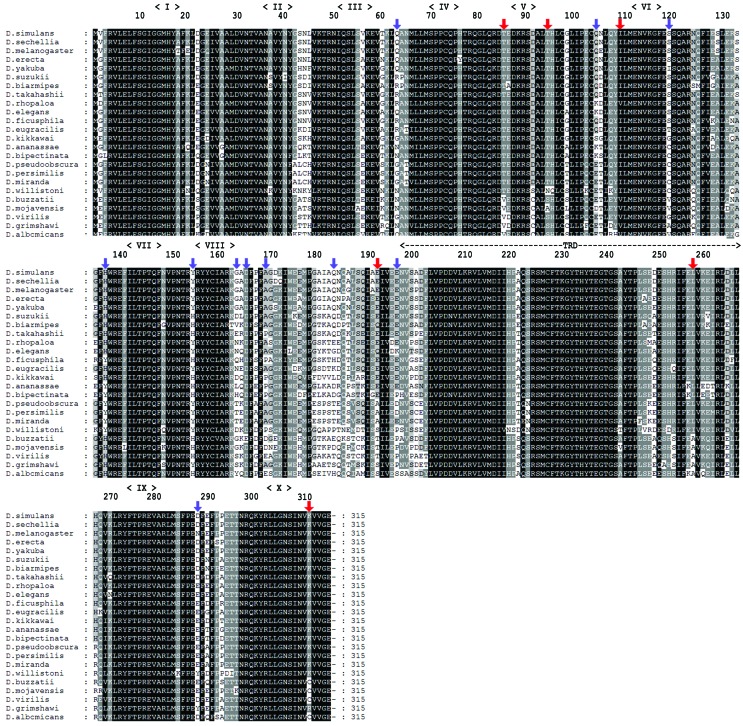
Multiple alignment of DNMT2 sequences used in the positive selection analysis with no gaps. Black boxes represent conserved 100% in all sequences, dark grey 80% and light grey 60%. Red arrows indicate sites under positive selection and purple arrows sites under positive-destabilizing selection. Roman letters indicate the conserved (cytosine-5) MT2 motifs. TRD indicates the target recognition domain.

**Figure 4:**
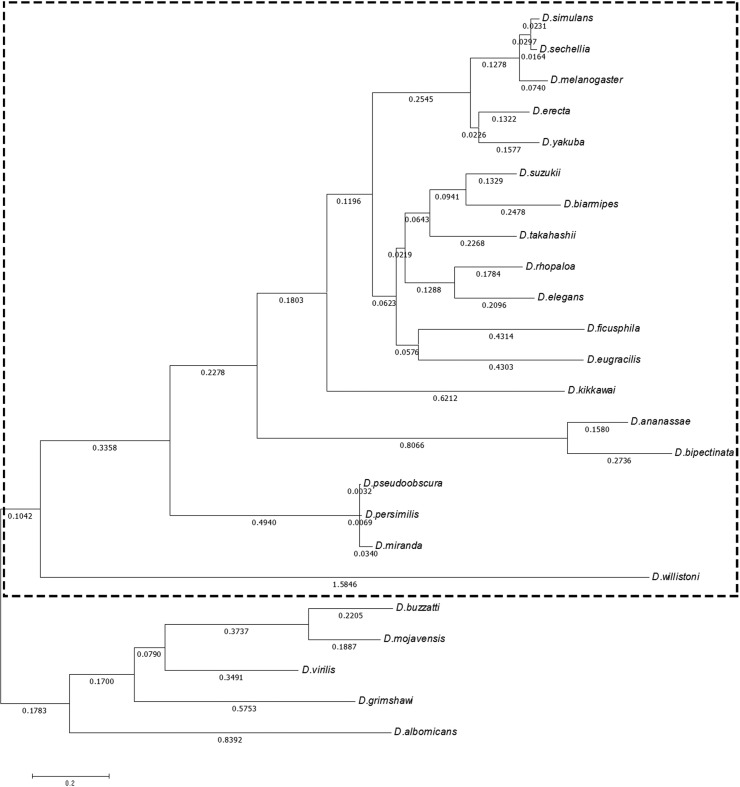
ML phylogenetic tree of *Dnmt2* used in analysis for detection of selection. The dotted frame highlights the *foreground* branch. The genetic distances are in nucleotide substitution per codon (number below the branches).

To evaluate positive selection in *Dnmt2*, we selected representative species of the main groups of drosophilids whose complete sequences could be obtained (Figure 3). Using the PAML package we obtained an initial tree that was later analyzed for positive selection in codeML. The initial ML tree recovers the evolutionary relationship between *Drosophila* and *Sophophora* subgenera (Figure 4), as well as the amino acid tree obtained from the Bayesian analysis (Figure 2). We used the results of the divergence analysis (Table 1) to establish which branch to use as *foreground*. When analyzing the results of divergence, we found that the divergence within the *Sophophora* subgenus is lower than the divergence between the *Sophophora* and *Drosophila* subgenera, as expected (Table S5). Moreover, the *Sophophora* and *Drosophila* subgenera show different evolutionary rates, with the *Sophophora* subgenus having more sites with fast evolution (Figure 1). Therefore, we asked whether the divergence observed between the groups is random or driven by selection. Thus, we established the *Sophophora* subgenus as *foreground* and the *Drosophila* subgenus as *background.*

The selective pressures over the *Dnmt2* sequence were investigated by the ratio of nonsynonymous to synonymous substitutions. Table 2 shows the parameters inferred for the null models (M1a and MA) and for the alternative MA model. The null model of selective constraint (0 < ω ≤ 1) was rejected by the relaxed branch-site test, indicating that the *foreground* branch (Figure 3) has diverged by relaxed selective constraint or by positive selection (Table 3). By the contrast of the restricted-MA versus MA models (*strict branch-site*), we could discriminate between the two hypotheses, where the null model of selective constraint was rejected (Table 3), in favor of the hypothesis that several sites of *Sophophora* DNMT2 (Figure 3 and Figure 4) differentiated by positive selection. We estimated that six sites (position 85, 94, 109, 192, 257 and 311) were evolving under positive selection (Table 3) (Figure 3).

**Table 2:** Parameters estimates and log likelihood values for branch-site M1a, MA and restricted MA model.

Model	Parameters			lnL
M1a	ω0 = 0.04651	p0 = 0.86743		-11 046.650097
*Relaxed branch-site test null model*	ω1 = 1.00000	p1 = 0.13257		
Restricted MA (ω2 = 1 fixed)	ω0 =0.04520	ω2 = 1.000		-11 039.638028
*Strict branch-site test null model*	p0 = 0.82646	p1 = 0.12587	(p2a + p2b) = 0.04767	
MA	ω0 = 0.04596	ω2 = 50.90099		-11 032.575669
*Alternative model*	p0 = 0.84965	p1 = 0.12542	(p2a + p2b) = 0.02493	

Lnl = log likelihood; ω0 = dN/dS values for sites with 0 < ω < 1; ω1 = dN/dS values fixed to 1; ω2 = dN/dS values for sites with ω > 1, which corresponds only to sites in the foreground branch.

**Table 3:** Comparison of null and alternative models by LRT and positively selected sites estimated by Bayes Empirical Bayes.

Test	Contrast	LRT	D.F.	χ2- Probability	Positively selected sites
*Relaxed branch-site*	M1a MA	25.20	2	*p* < 7.7x10^-7^	
*Strict branch-site*	Restricted MA MA	12.41	*1*	*p* < 0.001	85, 94, 109, 192, 257, 311

LRT - likelihood ratio test; D.F - Degrees of freedom.

**Figure 5:**
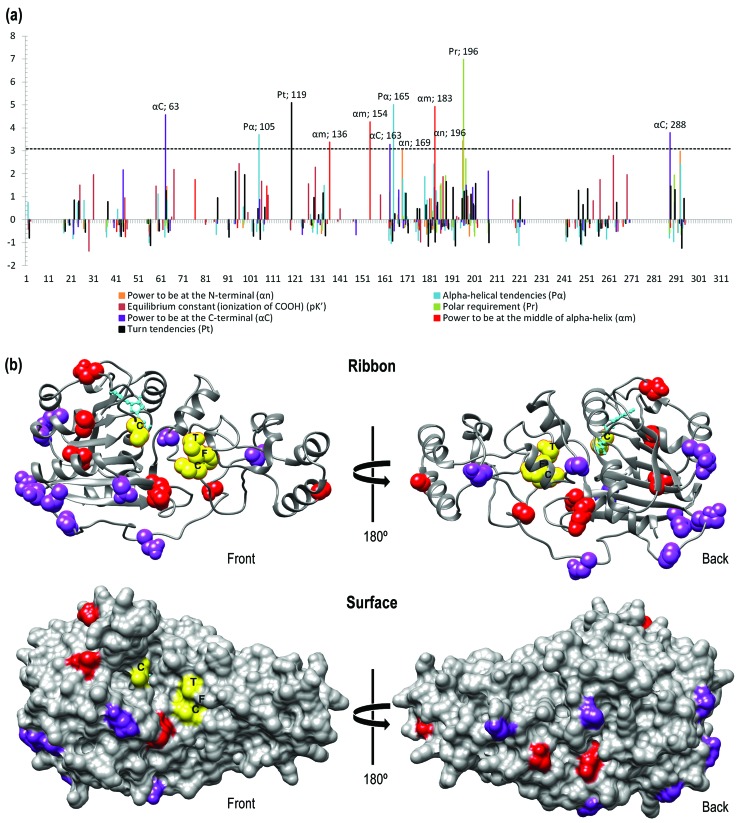
DNMT2 structure predictions. (a) Sliding window plots of the z-scores of radically changed properties showing regions under positive-destabilizing selection in *Dnmt2*. Dashed horizontal line indicates the Bonferroni-corrected significant limit (z-score = 3.09, *p* < 0.001). Alpha-helical tendencies (*P*_*a*_) – red; Equilibrium constant (ionization of COOH) (*pK’*) – orange; Polar requirement (*P*_*r*_) – green; Power to be at the C-terminal (*α*_*C*_) – blue; Power to be at the middle of alpha-helix (*α*m) – pink; Power to be at the N-terminal (*α*n) – black; Turn tendencies (*Pt*) – purple. (b) Structure of *sf*DNMT2 (PDVB: 4H0N) (Li *et al.*, 2012), ribbon (up) and surface (down) representations. The respective sites under positive selection in drosophilids are represented in *Spodoptera frugiperda* structure. Sites under positive selection colored red, positive-destabilizing selection purple, motif CFT from TRD and the catalytic cysteine yellow and cofactor s-adenosyl methionine (SAM) cyan.

To investigate selection on amino acid properties we used the software *TreeSAAP*, based on the global goodness-of-fit statistics calculated by the *MM01* method. All physicochemical properties examined are significant (cutoff = 0.05) (Table 4). Seven properties demonstrated significantly positive z-scores under a trait of radical changes category between 6 and 8: α-helical tendencies (*P*α), Equilibrium constant (ionization of COOH) (*pK’*), Polar requirement (*Pr*), Power to be at the C-terminal (*α*C), Power to be at the middle of alpha-helix (*α*m), Power to be at the N-terminal (*α*n) and Turn tendencies (*Pt*). Specific analysis with the sliding window in *TreeSAAP* showed that 12 amino acids were under positive-destabilizing selection (Figure 5a); the properties of most of these are related to the alpha-helix structures, mainly located in the catalytic domain.

**Table 4:** Amino acid physicochemical properties under positive destabilizing selection in DNMT2.

Physicochemical property	Goodness-of-fit (neutral expectation)	radical change category (6, 7 and 8)	z-score
Alpha-helical tendencies (*P*_*a*_)	33.339***	8	4.292***
Average number of surrounding residues (*N*_*s*_)	119.136***		6.442***
Beta-structure tendencies (*P*_*b*_)	30.909***		2.804**
Bulkiness (*B*_*l*_)	35.052***		3.104***
Buriedness (*B*_*r*_)	54.407***		2.580**
Chromatographic index (*R*_*f*_)	107.109***		3.190***
Coil tendencies (*C*_*t*_)	18.839**		-
Composition (*C*)	43.179***		3.898***
Compressibility (*K* ^0^)	22.548**		-
Equilibrium constant (ionization of COOH) (*pK’*)	79.39***	8	2.828**
Helical contact area (*C*_*a*_)	81.079***		3.732***
Hydropathy (*H*)	83.046***		3.885***
Isoelectric point (*pH*_*i*_)	45.283***		4.261***
Long-range non-bonded energy (*E*_*l*_)	78.459***		5.712***
Mean r.m.s. fluctuation displacement (*F*)	117.708***		5.736***
Molecular volume (*M*_*v*_)	77.479***		3.151***
Molecular weight (*M*_*w*_)	69.846***		3.577***
Normalized consensus hydrophobicity (*H*_*nc*_)	57.459***		2.062*
Partial specific volume (*V* ^0^)	61.97***		2.986**
Polar requirement (*P*_*r*_)	29.801***	7	2.341**
Polarity (*P*)	59.713***		2.522**
Power to be at the C-terminal (*α*_*C*_)	118.516***	6	5.758***
Power to be at the middle of alpha-helix (*α*_*m*_)	49.271***	7	3.657***
Power to be at the N-terminal (*α*_*n*_)	32.449***	7	3.159***
Refractive index (*μ*)	47.548***		3.632***
Short and medium range non-bonded energy (E_sm_)	67.785***		3.743***
Solvent accessible reduction ratio (R_a_)	83.159***		2.942**
Surrounding hydrophobicity (*H*_*p*_)	69.397***		2.345**
Thermodynamic transfer hydrohphobicity (*H*_*t*_)	50.917***		2.785**
Total non-bonded energy (*E*_*t*_)	109.548***		5.654***
Turn tendencies (*P*_*t*_)	109.136***	6	6.198***

* p < 0.05

** p < 0.01

*** p < 0.001

The DNMT2 sites that are under positive selection and positive-destabilizing selection (as indicated by the CodeML and TreeSAAP analyses, respectively) are shown in Figure 5b. Most sites under selection are located in the catalytic domain and especially located on the molecular surface. The only crystallographic model of DNMT2 available for arthropods is from *S. frugiperda* and it is used just as a representative model only (Li *et al.*, 2012).

### Protein-protein interactions and co-evolutionary predictions

We accessed the predicted genes as interacting with *D. melanogaster, D. ananassae*, *D. grimshawi*, *D. pseudoobscura*, *D. virilis*, *D. willistoni* and *D. yakuba Dnmt2* through the STRING database (Table 5). These were concatenated and the *D. melanogaster* gene annotations to ERC analysis were used, since the taxonomic group utilized in ERC Analysis Webserver is *D. melanogaster*. We also made an extensive search for genes that have significant ERC values with *D. melanogaster Dnmt2* by ERC Analysis Webserver Top Genes tool, which retrieves the genes with the highest ERC values for a given query gene from the entire genome (Clark *et al.*, 2012., 2013, Findlay *et al.*, 2014).

**Table 5:** Gene annotation and sequence location from STRING and ERC webserver searching from *D. melanogaster* genome. Genes with high ERC value (> 0.400) and p-value < 0.05 are colored gray.

Gene	Annotation	Description
Atac3	CG32343	Contributes to histone acetyltransferase activity. Involved chromatin remodeling.
Cap	CG18408	Interacts selectively and non-covalently with vinculin, a protein found in muscle, fibroblasts, and epithelial cells that binds actin and appears to mediate attachment of actin filaments to integral proteins of the plasma membrane.
CG10262	CG10262	Proliferating Cell Nuclear Antigen (PCNA) domain. These polymerase processivity factors play a role in DNA replication and repair.
CG13035	CG44836	Uncharacterized protein involved in sensorial perception of pain.
CG14618	CG14618	Belongs to the class IV-like SAM-binding methyltransferase superfamily. tRNA (guanine(9)-N(1))-methyltransferase TRM10. Enzyme catalyzes the conversion of a guanosine residue to N1-methylguanine in position 37, next to the anticodon, in tRNA.
CG14906	CG14906	N-6 adenine-specific DNA methylases. Specifically methylate the amino group at the C-6 position of adenines in DNA.
CG16863	CG16863	The protein has zinc finger, BED-type, which is thought to be involved in chromatin insulation at BEAF (boundary element-associated factor) and DREF, a transcriptional regulator.
CG17124	CG17124	PKC-activated protein phosphatase-1 inhibitor. Stops, prevents or reduces the activity of a protein phosphatase, an enzyme that hydrolyzes phosphate groups from phosphorylated proteins.
CG6712	CG6712	Probably RNA binding inferred from sequence or structural similarity with *Saccharomyces* RPF1, involved in ribosome biogenesis. Belongs to Brix superfamily.
CG7470	CG7470	Predicted gamma-glutamyl phosphate reductase with delta1-pyrroline-5-carboxylate synthetase activity, glutamate 5-kinase activity. An inner mitochondrial membrane enzyme, is essential to the de novo synthesis of the amino acids proline and arginine. Involved in epithelium development and germarium-derived egg chamber formation.
CTCF	CG8591	CTCF is a ubiquitous transcription factor that binds to insulators and domain boundaries. It mediates insulator function and blocks enhancers by binding to Cp190. It contributes to long-range chromatin interaction, organizes chromatin domain boundaries and coordinates nuclear architecture.
Dnmt2	CG10692	Methyltransferase 2 is a (cytosine-5) DNA/tRNA methyltransferase. It is involved in regulation of gene expression by cytosine-5 methylation. The major protein role is the modifications that protects tRNAs against endonucleolytic cleavage and contributes to stress resistance, protein translation and small RNA-mediated gene regulation.
Eggless	CG12196	Belongs to the class V-like SAM-binding methyltransferase superfamily. Histone-lysine methyltransferase family. Involved in negative regulation of gene expression.
Eno	CG17654	Responsible for the catalysis of the conversion of 2-phosphoglycerate (2-PG) to phosphoenolpyruvate (PEP), the ninth and penultimate step of glycolysis
Haywire	CG8019	Helicase Ercc3, core RNA polymerase binding transcription factor activity. Involved in regulation of alternative mRNA splicing, via spliceosome, cell proliferation and growth.
Homer	CG11324	Involved in the positive regulation of circadian sleep/wake cycle, sleep; response to ethanol, behavioral response to ethanol, regulation of locomotion and adult behavior. Activity in stress response.
hop	CG1594	Signal transduction-non receptor tyrosine kinase. Members of the Janus kinase (JAK) family of cytoplasmic protein tyrosine kinases physically associate with ligand-bound receptors. The JAK-STAT pathway regulates the expression of pair rule gene even-skipped (a transcriptional repressor of a number of genes) early in embryogenesis.
Hsp22	CG4460	A key player in cell-protection mechanisms against oxidative injuries and aging in Drosophila. Activated in late third-instar larvae of Drosophila melanogaster in the absence of heat stress
MBD-like	CG8208	Methyl Binding Protein 2/3, a co-repressor and an integral component of the nucleosome remodelling and deacetylase (NuRD) complex. Negative regulation of transcription, involved in organism development
mei-S332	CG5303	Acts to maintain sister-chromatid cohesion before anaphase II of meiosis in both males and females.
mms4	CG12936	Methyl methanesulfonate sensitivity 4 is the non-catalytic subunit of the mus81-mms4 structure-selective endonuclease that functions in DNA repair.
mus209	CG9193	Belongs to the PCNA family. Involved in eggshell chorion gene amplification, DNA replication, mismatch repair, neurogenesis and antimicrobial humoral response.
Nsun2	CG6133	tRNA (cytosine-5-)-methyltransferase activity.
Orc2	CG3041	Origin recognition complex subunit 2. It is involved in eggshell chorion gene amplification, chromatin silencing, cell proliferation, DNA replication initiation, chromosome condensation and neurogenesis.
Pnt	CG17077	It is a sequence-specific DNA binding transcription factor activity, repressing transcription factor binding, involved in positive regulation of transcription. It is involved in organism developmental process, open tracheal system development, post-embryonic organ morphogenesis, regulation of developmental process, sensory organ development, cardiovascular system development, regulation of RNA metabolic process, positive regulation of cell proliferation, anterior/posterior axis specification, multi-organism reproductive process, muscle cell differentiation, compound eye photoreceptor development, regulation of neurogenesis.
Rel	CG11992	Relish is a transcription factor and the downstream component of the Immune Deficiency pathway, which regulates the antibacterial response and other less characterized cellular processes.
RhoGEF4	CG8606	Rho family small GTPases act as molecular switches that regulate neuronal morphogenesis, including axon growth and guidance, dendritic spine formation, and synapse formation. These proteins are positively regulated by guanine nucleotide exchange factors (GEFs) of the Dbl family. Findings suggest that DRhoGEF4 may participate in cytoskeleton-related cellular events by specifically activating RhoA in neuronal morphogenesis.
Rpd3	CG7471	Histone deacetylase 1. Catalyzes the deacetylation of lysine residues on the N-terminal part of the core histones (H2A, H2B, H3 and H4). Histone deacetylation may constitute a tag for epigenetic repression and plays an important role in transcriptional regulation, cell cycle progression and developmental events. For instance, deacetylation of histone H3 may be a prerequisite for the subsequent recruitment of the histone methyltransferase Su(var)3-9 to histones. Involved in position-effect variegation (PEV).
Rpp20	CG33931	A subunit of the RNase P and RNase MRP holoenzymes, has interaction with the Drosophila SMN protein. Immunofluorescence results indicate that Rpp20 is diffusely distributed throughout the cytoplasm with higher concentration observed in the nucleus. However, in response to stress, SMN forms aggregates and redistributes Rpp20 into punctuated cytoplasmic SMN granules.
Sna	CG3956	Snail is a transcription factor that contributes to embryonic mesoderm development, epithelial to mesenchymal transition and asymmetric cell division.
Su(var)2-5	CG8409	Suppressor of variegation 205 is a heterochromatin protein associated with the pericentric heterochromatin and telomeres in Drosophila. It is involved in the positive autoregulatory expression and can bind directly to nucleosomes.
Su(var)2-10	CG8068	It is responsible for establishing and maintaining chromosome organization in interphase nuclei, promoting chromosome structure and function.
Su(var)3-3	CG17149	Probable histone demethylase that specifically demethylates ‘Lys-4’ of histone H3, a specific tag for epigenetic transcriptional activation, thereby acting as a corepressor. Required for heterochromatic gene silencing
Su(var)3-9	CG43664	Histone methyltransferase that specifically trimethylates ‘Lys-9’ of histone H3 using monomethylated H3 ‘Lys-9’ as substrate. H3 ‘Lys-9’ trimethylation represents a specific tag for epigenetic transcriptional repression by recruiting Su(var)205/HP1 to methylated histones. Mainly functions in heterochromatin regions, thereby playing a central role in the establishment of constitutive heterochromatin at pericentric regions. Involved in heterochromatic gene silencing including the modification of position-effect-variegation.
Tet	CG9973	Ten-Eleven Translocation (TET) family protein. Involved in positive regulation of DNA demethylation, inducing positive regulation of transcription from RNA polymerase II promoter.
Thor	CG8846	Eukaryotic translation initiation factor 4E binding protein, controlled by tor. It contributes to translation regulation, response to environmental stress and cell growth regulation.

Altogether, 551 genes were obtained with an ERC value > 0.400 (p-value < 0.05) as result of the search. We filtered the genes found according to the expression period from 00-12 hour of the embryonic stage, which corresponds to the expression of *Dnmt2* in *D. melanogaster* according to Lyko *et al.* (2000). The biological role played by genes according to the biological process described for DNMT2 was also considered (i.e., epigenetic functions, transcriptional control, stress response, tRNA methylation, response to heat, positive regulation of innate immune response, telomere maintenance). At the end, we obtained a list of 35 genes and added the enolase enzyme, since this was the first DNMT2 interacting protein described (Tovy *et al.*, 2010) (Table 5).

ERC values for the 36 genes were obtained by the Group ERC Analysis tool, which returns the ERC values between a group of genes and statistics for the strength of ERC between them (Table S6). Interestingly, *Dnmt2* presents high ERC values related with 10 genes (p < 0.05) (Table 6), in which four are related to transcriptional control and three have a chromatin-remodeling function. However, genes with protein interaction prediction coming from the STRING database showed low ERC values (Table 6). The 15 genes with an ERC value > 0.400 (p-value < 0.05) are distributed between the Muller’s elements A, B, C, D and E.

**Table 6:** ERC values between potential DNMT2 protein-protein partners. The matrix shows all pairwise ERC values between genes below the diagonal and respectively p-values above the diagonal. Cells are shaded red according to the intensity of their deviation from the null expectation.

Protein		1	2	3	4	5	6	7	8	9	10	11	12	13	14	15	16	17	18
1	pnt	N/A	0.0010	0.1445	0.0781	0.4435	0.1082	0.0950	0.1429	0.3571	0.1486	0.1924	0.3492	0.0145	0.4435	0.0207	0.0170	0.0241	0.0760
2	Su(var)2-10	0.811	N/A	0.0578	0.0168	0.0143	0.0868	0.0891	0.0912	0.1791	0.1679	0.3720	0.5330	0.0185	0.0912	0.0493	0.0572	0.0383	0.2213
3	CG14618	0.375	0.480	N/A	0.0093	0.0398	0.0541	0.0117	0.0249	0.0220	0.1015	0.1111	0.1780	0.0123	0.0732	0.0489	0.1203	0.0775	0.4843
4	CG14906	0.473	0.597	0.731	N/A	0.0089	0.0125	0.0025	0.0008	0.0387	0.0459	0.0879	0.2154	0.0266	0.1378	0.1770	0.1251	0.0431	0.4492
5	CG32343	0.000	0.608	0.593	0.782	N/A	0.0032	0.0070	0.0057	0.1500	0.0492	0.1193	0.3912	0.0180	0.0818	0.2100	0.1325	0.1437	0.3912
6	CTCF	0.426	0.429	0.555	0.758	0.801	N/A	0.0004	0.0032	0.0435	0.0238	0.1094	0.1260	0.0641	0.1074	0.1074	0.1087	0.0153	0.1688
7	CG16863	0.447	0.424	0.713	0.844	0.763	0.932	N/A	0.0009	0.0304	0.0262	0.0448	0.0905	0.0267	0.0693	0.0804	0.0794	0.0165	0.2525
8	sna	0.377	0.421	0.650	0.874	0.773	0.833	0.912	N/A	0.0149	0.1107	0.0146	0.0721	0.0278	0.0655	0.0518	0.0411	0.0127	0.3520
9	mei-S332	0.087	0.311	0.662	0.650	0.386	0.643	0.698	0.796	N/A	0.1072	0.0903	0.0312	0.0248	0.4685	0.0943	0.1754	0.0643	0.5163
10	homer	0.370	0.323	0.457	0.622	0.587	0.708	0.713	0.524	0.457	N/A	0.0026	0.0788	0.0196	0.1536	0.1112	0.0661	0.0397	0.0903
11	Rpp20	0.307	0.109	0.441	0.513	0.440	0.506	0.651	0.797	0.489	0.777	N/A	0.0127	0.0186	0.1018	0.0495	0.0950	0.0703	0.1915
12	RhoGEF4	0.097	-0.025	0.337	0.313	0.000	0.480	0.541	0.602	0.638	0.498	0.716	N/A	0.0322	0.0241	0.0250	0.1788	0.1488	0.1462
13	Mt2	0.644	0.590	0.711	0.693	0.699	0.589	0.712	0.741	0.663	0.650	0.680	0.577	N/A	0.0179	0.0049	0.0339	0.0392	0.1800
14	Orc2	0.000	0.421	0.512	0.425	0.509	0.508	0.585	0.616	0.000	0.375	0.470	0.609	0.686	N/A	0.0044	0.0428	0.4939	0.1165
15	hay	0.609	0.497	0.572	0.369	0.297	0.508	0.563	0.653	0.481	0.440	0.582	0.605	0.771	0.741	N/A	0.0028	0.0082	0.0493
16	mms4	0.631	0.481	0.426	0.445	0.418	0.507	0.565	0.686	0.344	0.521	0.483	0.313	0.622	0.542	0.831	N/A	0.0041	0.0273
17	hop	0.596	0.524	0.503	0.629	0.399	0.747	0.762	0.805	0.540	0.588	0.528	0.351	0.602	0.000	0.774	0.845	N/A	0.0940
18	mus209	0.476	0.264	0.020	0.052	0.000	0.409	0.295	0.185	-0.010	0.479	0.336	0.355	0.349	0.397	0.621	0.703	0.551	N/A

## Discussion

### Evolutionary scenario of drosophilids DNMT2

In our analyses of *Dnmt2* homologs among species of Drosophilidae (Dot blot, Figure S1), we detected a hybridization signal in the majority of the analyzed species. Not all species amplified using the *D. melanogaster* and *D. willistoni* primers, indicating that the degree of *Dnmt2* similarity among Drosophilidae species varies (Table S1). The representative species of the *Dorsilopha* subgenus did not present *Dnmt2* amplification with the primers used, which could indicate that the 5’ and 3’ regions of the *Dnmt2* gene must have certain variation in the species that did not show amplicons (Table S1). However, the average divergence value did not exceed 22.5% for the amino acid sequences, or 26.57% for the nucleotide sequences (data not shown). Functionally important genes are often evolutionarily constrained because the amino acid sequence must be preserved for a protein’s catalytic or structural role to be maintained (Figures 3 and S2).

Overall, the Neotropical *willistoni* species group presents the highest differences when compared to all other groups, both in the nucleotide and amino acid analysis (Table 1). Interestingly, the difference is reflected in the increased presence of basic amino acids in the DNMT2 of the *willistoni* subgroup. Such differences may result in changes in physicochemical properties of the enzyme, giving modulations and differential affinities between proteins of different groups. Shifts in codon preferences are described in *D. willistoni* (Singh *et al.*, 2006; Vicario *et al.*, 2007), and being a lineage-specific feature, it is suggested that the differential preference may influence the evolution of DNMT2 in the species of the *willistoni* group. Also, molecular evolution is atypical in this species of the *willistoni* group (and its sister group *saltans*), characterized by high rates of nucleotide substitution and low portions of G/C (DeSalle, 1992; Powell and DeSalle, 1995; Remsen and O’Grady, 2002).

Our phylogenetic analysis further showed that the DNMT2 relationships in Drosophilidae comprised three main clades: a *virilis-repleta* section, a *quinaria-tripunctata* section and the *Sophophora* subgenus (Figures 1 and S2), consistent with previous studies regarding the phylogeny of the genus (Lewis *et al.*, 2005; Robe *et al.*, 2005, 2010; Wang *et al.*, 2006; Hatadani *et al.*, 2009), although for the *quinaria-tripunctata* section, the evolutionary relationships between the species did not have a strong support value and presented, in some cases, polytomy. Monophyly of the subgenus *Sophophora* has been confirmed as well by Tatarenkov *et al.* (1999) and Robe *et al.* (2005). These findings suggest that the DNMT2 sequences exhibit similar patterns to the species evolution.

Despite the conservation of drosophilid DNMT2s, at least 10 regions have a high evolutionary rate along the protein (Figure 1). Noteworthy, region 7 has a long sequence with high evolutionary rate. The structural location of this region corresponds to the connecting handle between the catalytic domain and TRD. When analyzed separately, the evolutionary rates of the *Drosophila* and *Sophophora* subgenera showed very similar patterns with regard to the distribution of sites that evolve faster. Nonetheless, evolutionary rate values in the *Drosophila* subgenus seem to be smaller than those of *Sophophora* (Figure 1). Thus, we question whether some of these sites with faster evolutionary rates may be under some lineage-specific adaptive selection event, or just correspond to a relaxed selection pressure.

### Positively selected sites in DNMT2 suggest adaptation for protein-protein interactions

The importance of *Dnmt2* for development in drosophilid species remains unclear. Marhold *et al.* (2004) compared DNMT2 sequences from different dipterans, showing high evolutionary conservation mainly in the catalytic domain. The occurrence of DNA methylation in various dipteran species was also demonstrated, suggesting that DNMT2-mediated DNA methylation has a deep evolutionary relationship of at least 250 million years. In any case, DNMT2-mediated DNA methylation remains an open issue.

By depletion of *Dnmt2* with RNA interference, Kunert *et al.* (2003) demonstrated that DNMT2 is both necessary and sufficient for DNA methylation in *D. melanogaster* and suggest a different target sequence for DNA methylation: CpT/A. However, depletion of *Dnmt2* had no detectable effect on embryonic development, despite complete loss of DNA methylation. On the other hand, DNMT2 has low catalytic activity on DNA (Hermann *et al.*, 2003; Fisher *et al.*, 2004), and it seems to have a preference for tRNA-Asp as a methylation target (Goll *et al.*, 2006; Jurkowski *et al.*, 2008; Krauss and Reuter 2011; Tuorto *et al.*, 2012; Durdevic *et al.*, 2013a; Shanmugam *et al.*, 2014). More recently, Takayama *et al.* (2014) have brought another piece to the controversial and elusive puzzle of DNA methylation in the genome of *D. melanogaster*: lines deficient for DNMT2 retain genomic methylation, although with altered patterns. Adding up to this peculiar field, there is the phenomenon of sex-specific DNA methylation in species of the subgroup *willistoni* (Garcia *et al.*, 2007; D’Ávila *et al.*, 2010), which raises questions about the mechanisms that are directly involved in the sex-specific DNA methylation process in this drosophilid group.

These scenarios raise issues about the selective pressures acting on *Dnmt2* and on how the DNMT2 protein may have evolved along the several Drosophilidae lineages. Considering that *Dnmt2* was described as unnecessary for embryological development (Kunert *et al.*, 2003) and no loss of fitness with depletion of DNMT2 was detected (Takayama *et al.*, 2014) in *D. melanogaster*, one might expect a relaxed selective constraint. However, the present study demonstrated that *Dnmt2* is evolutionarily conserved (Figures 2 and S2), as previous studies have already shown (Marhold *et al.*, 2004), and therefore, it could be suggested that the entire gene has evolved under purifying selection and that positive selection has played only a minor role in the evolution of Drosophilidae *Dnmt2*.

Since the catalytic domain (residues from the cofactor-binding pocket and catalytic residues in the motifs ENV and PPC) and the motif CFT in TRD are generally conserved (Figures 3 and 5b), the 10 sites with high evolutionary rates in some motifs in the catalytic domain and most of the TRD motif would be due to relaxed selective constraint or neutral evolution (Figure 1). On the other hand, this variation would be better explained by an adaptive selection. Nevertheless, when we employed analyses which considered variation in ω rates in branches and sites, we found several positively selected sites in the catalytic domain and in the TRD from *Dnmt2* between *foreground* (subgenus *Sophophora*) and *background* (subgenus *Drosophila*) lineages (Table 3 and Figure 5b). Positive selection has been shown to act directly on amino acid residues exposed on the surface of proteins, while those in the core remain stable (Casewell *et al.*, 2011), favoring the maintenance of catalytic function of the protein.

The majority of sites under positive-destabilizing selection were located at the protein surface, which suggests that these sites are potentially involved in interactions with the surrounding environment. The sites 63, 163 and 288 (Figure 4a,b) were noted as having radically changed properties, namely Power to be at the C-terminal (αC). This property relates to the ability of residues to interact with other molecules, especially protein-protein and subunit interactions. Also, sites 136 to 183 are located in the connecting region of the catalytic domain and TRD and could be a potential region of protein interaction.

### DNMT2 has new potential partners that have not been considered yet

Tovy *et al.* (2010) established enolase as the first DNMT2 interacting protein and highlighted an unexpected role of a glycolytic enzyme in the modulation of DNMT2 activity. Other potential protein partners of DNMT2 have been described, such as EGGLESS (dSETDB1), which is a member of the family of SET/MBD proteins and methylates lysine 9 in histone H3, that mediates DNA methylation and is involved in silencing genes and retrotransposons (Gou *et al.*, 2010). Another probable candidate is MBD-like, a methyl-DNA binding protein that keeps certain genes epigenetically silenced during genome activation (Marhold *et al.*, 2004; Gutierrez and Sommer, 2004). Since DNMT2 has tRNA as a preferential target, NSUN2 (a tRNA methyltransferase which has the function of cytosine-C5 methylation) may also be a possible protein partner, cooperating with tRNA stability and protein synthesis (Tuorto *et al.*, 2012), together with DNMT2.

ERC values are typically elevated between interacting proteins and can be used to establish molecular and functional interactions between a pair and/or a group of proteins. Therefore, here we tested 36 proteins retrieved from searches through the STRING database and the tool ERC Analysis Webserver Top Genes. Interestingly, we found that the protein-interactions predicted with DNMT2 (STRING database) have low ERC values, mostly with negative values (Table 6). We had expected that high ERC values would be found in the protein pairs that are known to belong to epigenetic mechanisms predicted to interact with DNMT2. However, only the CG6712, MDB-like, RPD3 and SU(VAR)2-5 proteins showed positive ERC values, but these were very low (ERC < 0.3). DNMT2 and mus209 showed a low ERC (0.369) but this was not significant (p < 0.18). Even enolase, which was described as the first DNMT2 interacting protein (Tovy *et al.*, 2010), showed a low ERC value. Nonetheless, Clark *et al.* (2012) demonstrated that direct physical interaction is not required to establish a high evolutionary rate covariation, but that the two major components associated with ERC are cofunctionality (functional and physical interactions annotations) and the coevolution of expression levels. When we looked at the proteins with high ERC values, we observed a variety of proteins with different levels of gene expression regulation (Table 6). This was not surprising, given the previously known subtract duality and the diversity of biological processes involving DNMT2 (Kunert *et al.*, 2003; Tang *et al.*, 2003; Lin *et al.*, 2005; Goll *et al.*, 2006; Phalke *et al.*, 2009; Schaefer *et al.*, 2010; Durdevic *et al.*, 2013b). This said, we can rank the proteins with high ERC values into six groups: (1) chromatin remodeling, (2) transcription factors, (3) expression regulation, (4) DNA replication, (5) stress response, and (6) RNA editing.

The first group shows that DNMT2 has a high ERC value with proteins related to chromatin remodeling, like ATAC3 that is an essential *D. melanogaster* histone acetyl transferase (HAT) complex, together with transcriptional cofactors GCN5 (KAT2), ADA3, ADA2A, ATAC1 and HCF. This complex does not work in nucleosome remodeling itself, but it stimulates nucleosome sliding by the ISWI, SWI-SNF and RSC complexes (Suganuma *et al.*, 2008). The nucleosome remodeling process is required to expose sequences that may be a target for gene silencing.

In their review, Klose and Bird (2006) describe that in targeting *de novo* DNA methylation, transcription factors (TF) have the capacity to interact with DNMT enzymes and to promote cytosine-target methylation as a part of the molecular silencing repertoire. This specific DNMT-TF network may be responsible for promoting specific differentiation stages in different organs. The significant ERC values found between DNMT2 and TFs like PNT and SNAIL (Table 6) reinforce the DNMT-TF interaction findings.

The involvement of DNMT2 in gene expression regulation appears in the interaction with histone methyltransferases enzymes (HMT) with high ERC values between the heterochromatin protein (HP) SU(VAR)2-10. SU(VAR)2-10 encodes a member of the PIAS protein family that controls diverse functions and is involved in different aspects of chromosome structure and function by establishing/maintaining chromosome organization (Hari *et al.*, 2001). Furthermore, DNMT-TF interaction and DNMT-HP interaction can produce a ternary complex composed of a DNMT, a MBD and an HP, promoting the recruitment of histone H3K9 methyltransferases (HMTs) (Rai *et al.*, 2010). The CG16863 protein has a BED-type zinc finger domain and is not characterized yet. BED fingers are able to bind DNA and are present in some proteins like *Drosophila* boundary element-associated factor (BEAF), responsible by chromatin insulation and also required during early development (Gilbert *et al.*, 2006). DREF (DNA replication-related element-binding factor) is another protein that contains BED fingers; it is a transcription regulatory factor and it interacts genetically and physically with regulatory factors related to chromatin structures. Matsukage *et al.* (2008) identified more than 150 genes carrying DRE sequences in their promoter regions, most of them related to DNA replication, transcriptional regulation, cell cycle regulation, growth signal transduction and protein metabolism. The high ERC value between DNMT2 and BED-finger domain (0.712) is very interesting (Table 6), because the DREF target sequence is 5’-TATCGATA-3’, carrying a CpG motif that can be a DNMT2 target for cytosine methylation.

Another protein group that shares a high ERC with DNMT2 is related with expression regulation: HOPSCOTCH and HAYWIRE. HOPSCOTCH is a protein pseudokinase and is involved in many biological processes, like cell proliferation, structure morphogenesis, and others. Hou *et al.* (1996) have described Hopscotch/JAK kinase as an invertebrate JAK/STAT system. HOPSCOTCH regulates the transcription of target genes, such as the pair-rule gene *even-skipped*. *even-skipped* is a transcriptional repressor of a number of genes during early embryogenesis. Thus, having its role in the epigenetic system may DNMT2 have coevolved with Hopscotch/JAK to rearrange the epigenetic marks during the early development of drosophilids.

We also found significant evolutionary rate covariation values with proteins involved in DNA replication like ORC2, MMS4 and even MUS209 (a Proliferating cell nuclear antigen – PCNA) (Table 6). This may be linked to DNMT2 maintenance methylation of DNA in the replication event, or to *de novo* methylation during embryonic development.

Usually present in the nucleus, under conditions of stress, DNMT2 reallocated to cytoplasmic stress granules and RNA processing bodies (P-bodies). Thiagarajan *et al.* (2011) describe DNMT2 as part of the RNA processing machinery during cellular stress. During heat shock conditions, DICER-2 degrades tRNA and tRNA fragments, so DNMT2 can limit the extent of tRNA fragmentation during a stress event, since long double-stranded RNAs (dsRNAs) inhibit DICER-2 activity. Hence, DNMT2 is essential for DICER-2 processing in *Drosophila* (Durdevic *et al.*, 2013a).

Another role of DNMT2 against stress events is the silencing of retrotransposons and the control of RNA viruses in *Drosophila* (Phalke *et al.*, 2009; Durdevic *et al.*, 2013b). So, DNMT2 seems to be important as a control tool to various forms of stress that involve RNA, which can be triggered by an excess of endogenous (retrotransposons) or exogenous (viruses) RNAs. The presence of the proteins HOMER and Rpp20, with high ERC values, may contribute to the presence and function of DNMT2 involved in the response to stress events and opens new possible partner proteins. HOMER protein acts in response to ethanol (Urizar *et al.*, 2007), controlling circadian cycles (Naidoo *et al.*, 2012), and acts during the stress response. Rpp20 is a subunit of RNase P and RNase MRP that is involved in precursor rRNA processing (Li *et al.*, 2002) and interacts with SMN protein in response to stress (Hua and Zhou 2004).

Finally, we denoted an evolutionary rate relationship between DNMT2 and two nucleotide modification enzymes: CG14906 e CG14618. The CG14906 gene product corresponds to a TM-A70-like factor, an mRNA (2-O-methyladenosine-N(6)-)-methyltransferase (Bujnicki *et al.*, 2002), and CG14618 belongs to the class IV-like SAM-binding methyltransferase superfamily, tRNA methyltransferase TRM10 family. TRM10 catalyzes all known instances of m^1^G9 modification and, according to Swinehart *et al.* (2013), it is involved in different pathways beyond tRNA processing. The plurality and importance of RNA and tRNA modifications found in the last decade are well described (Chiari *et al.*, 2010; Chan *et al.*, 2012; Gu *et al.*, 2014; Hori, 2014; Swinehart and Jackman., 2015; Tuorto *et al.*, 2015). The pathway diversity and response plasticity to environmental modifications makes the tRNA/RNA processing enzymes a major link between the genome and environment.

## Conclusions

Our study shows that, although *Dnmt2* is highly conserved within the Drosophilidae family, it carries several nonsynonymous changes in some domains, which were shown to be maintained by positive and destabilizing selection. Purifying selection remains the major maintenance process of gene function(s), but positive selection appears to act on certain domains potentially involved with environment interactions. Thus, the findings suggest that the residues affected by positive selection may be involved in an interaction-driven co-evolution and the connection of regions of catalytic domains and TRD that probably would interact – direct or indirectly – with other proteins.

We suggest that the episodes of adaptive evolution in *Dnmt2* could be related to the wide diversity of niches, behaviors, amplitude distribution of drosophilids, as well as with and other peculiarities, such as the presence of transposable elements, chromosomal inversions, chromosomal stability, sex-specific DNA methylation, responsive modulation of RNA methylation (coding and noncoding), and endosymbiotic interactions. The multiplicity of proteins having strong evolutionary rate covariation found in the present work supports the hypothesis of a plurality of DNMT2 functions in *Dnmt2*-only organisms, like drosophilids. Since epigenetic systems are dynamic and change with the environment and along the evolutionary history of the organisms, we think that a wide scenario was opened, and the next step will be to analyze probable *Dnmt2* interactions with other genes along the evolution of different lineages in their ecological and evolutionary contexts.

## Supplementary material

The following online material is available for this article:

Table S1 - Drosophilidae species used in the present study.

Table S2 - Complete MT2 homologous sequences annotation.

Table S3 - GenBank accession numbers for nucleotide sequences obtained by DNA sequencing.

Table S4 - Standard error estimate(s) from estimates of evolutionary divergence between sequence pairs of different Drosophilidae species groups.

Table S5 - Estimates of Average Percentual Divergence over Sequence Pairs Groups.

Table S6 - ERC values for 36 genes obtained by Group ERC Analysis tool and STRING database.

Figure S1 - Dot blot screening for Dnmt2 sequences homologous to the *Drosophila melanogaster Dnmt2* gene.

Figure S2 - Multiple sequence alignment of DNMT2 showing the high conservation among *Drosophila* species.

Figure S3 - Bayesian phylogenetic analysis of *Dnmt2* using nucleotide sequences.
