# Supplementary material for: Evolution of DNMT2 in drosophilids: Evidence for positive and purifying selection and insights into new protein (pathways) interactions
Source: Genet Mol Biol. 2018 Mar 26;41(1 Suppl 1):215–34. doi: 10.1590/1678-4685-GMB-2017-0056 (PMC5913717; doi:10.1590/1678-4685-GMB-2017-0056)
Supplement: Supplementary file 1 [file 1415-4757-GMB-41-01-2017-0056-s001.pdf]

Supplementary Material to "Evolution of DNMT2 in drosophilids: Evidence for positive and purifying selection and insights into new protein (pathways) interactions"

Table S1 - Drosophilidae species used in the present study. Results from Dot blot and PCR screening are shown, as well as the species when the sequences used in this work were obtained.

Genus *Drosphila*					Genus *Sophophora*				
Group	Species	Dot	PCR	Sequence	Group	Species	Dot	PCR	Sequence
guarani	*D. ornatifrons*	+	+	√	*melanogaster*	*D. melanogaster*	+	+	**DB**
	D. subbadia	+	+	√		*D. simulans*	+	+	**DB**
	D. guaru	+	+	√		*D. sechellia*	**NT**	+	**DB**
guaramunu	*D. griseolineata*	+	+	√		*D. mauritiana*	+	+	√
	D. maculifrons	+	+	√		*D. teissieri*	+	+	√
tripunctata	*D. nappae*	+	+	√		*D. santomea*	+	+	√
	D. paraguayensis	**NT**	-			*D. erecta*	+	+	**DB**
	D. crocina	+	+	√		*D. yakuba*	+	+	**DB**
	D. paramediostriata	+	-			*D. kikkawai*	+	-	**DB**
	D. tripunctata	+	+	√		*D. ananassae*	+	+	**DB**
	D. mediodiffusa	**NT**	+	√		*D. bipectinata*	**NT**	**NT**	**DB**
	D. mediopictoides	+	-			*D. malerkotliana*	+	-	
cardini	*D. neocardini*	+	-			*D. orena*	+	-	
	D. polymorpha	+	-			*D. ficusphila*	**NT**	**NT**	**DB**
pallidipennis	*D. pallidipennis*	**NT**	-			*D. takahashii*	**NT**	**NT**	**DB**
calloptera	*D. ornatipennis*	+	+	√		*D. rhopaloa*	**NT**	**NT**	**DB**
immigrans	*D. immigrans*	+	+	√		*D. suzukii*	**NT**	**NT**	**DB**
	D. albomicans	**NT**	**NT**	**DB**		*D. biarmipes*	**NT**	**NT**	**DB**
funebris	*D. funebris*	+	-			*D. eugracilis*	**NT**	**NT**	**DB**
mesophragmatica	*D. gasici*	+	+	√		*D. elegans*	**NT**	**NT**	**DB**
	D. brncici	**NT**	-		*obscura*	*D. pseudoobscura*	+	+	**DB**
	D. gaucha	+	+	√		*D. miranda*	**NT**	**NT**	**DB**
	D. pavani	**NT**	+	√		*D. persimilis*	**NT**	**NT**	**DB**
repleta	*D. hydei*	+	+	√	*saltans*	*D. prosaltans*	+	-	
	D. mercatorum	+	-			*D. saltans*	+	-	
	D. mojavensis	+	+	**DB**		*D. neoelliptica*	+	-	
	D. buzzatii	**NT**	-	**DB**		*D. sturtevanti*	+	-	
canalinea	*D. canalinea*	+	-		*willistoni*	*D. sucinea*	+	**NT**	
flavopilosa	*D. cestri*	**?**	-			*D. nebulosa*	+	-	
	D. incompta	+	+	√		*D. willistoni (Gd-H4)*	+	+	**DB**
virilis	*D. virilis*	+	+	**DB**		*D. willistoni (Wip-4)*	+	**NT**	
grimshawi	*D. grimshawi*	**NT**	**NT**	**DB**		*D. willistoni (17A2)*	+	**NT**	
robusta	*D. robusta*	+	-			*D. paulistorum*	+	-	
*busckii****	*D. busckii****	+	-			*D. insularis*	+	-	
	*Z. indianus**	+	-			*D. tropicalis*	+	+	√
	*Z. tuberculatus**	+	-			*D. equinoxialis*	+	-	
	*S. latifasciaeformis***	+	-			*D. capricorni*	+	-	
	*S. lebanonensis***	+	-			*D. fumipennis*	+	-	

(+) signal detected in Dot blot or PCR amplification; (-) no PCR-specific amplification; (NT) not tested; (√) Dnmt2 homologous sequence obtained by PCR; (DB) Dnmt2 homologous sequence obtained via data bank (Genome/GenBank)

**Zaprionus* genus: *Z. indianus* and *Z. tuberculatus*

***Scaptodrosophila* genus: *S. latifasciaeformis* and *S. lebanonensis*

****Dorsilopha* subgenus: *D. busckii*
