# Supplementary material for: Evolution of DNMT2 in drosophilids: Evidence for positive and purifying selection and insights into new protein (pathways) interactions
Source: Genet Mol Biol. 2018 Mar 26;41(1 Suppl 1):215–34. doi: 10.1590/1678-4685-GMB-2017-0056 (PMC5913717; doi:10.1590/1678-4685-GMB-2017-0056)
Supplement: Supplementary file 2 [file 1415-4757-GMB-41-01-2017-0056-s002.pdf]

Supplementary Material to "Evolution of DNMT2 in drosophilids: Evidence for positive and purifying selection and insights into new protein (pathways) interactions"

Table S2 - Complete MT2 homologous sequences annotation.

	Annotation symbol
Drosophila albomicans	gi|405988621|gb|JH859534.1| Drosophila albomicans strain KM55-5 unplaced genomic scaffold Dalb_scaffold_67169, whole genome shotgun sequence
Drosophila ananassae	gnl|dana|scaffold_12943 type=golden_path_region; loc=scaffold_12943:1..5039921; ID=scaffold_12943; dbxref=GB:CH902624
Drosophila biarmipes	gi|459197679|gb|KB462833.1| Drosophila biarmipes unplaced genomic scaffold scf7180000302422, whole genome shotgun sequence
Drosophila bipectinata	gi|459198809|gb|KB464388.1| Drosophila bipectinata unplaced genomic scaffold scf7180000396728, whole genome shotgun sequence
Drosophila buzzatii	lcl|scaffold27 [847237 - 848191] and [847126 - 847185]
Drosophila elegans	gi|459200570|gb|KB458413.1| Drosophila elegans unplaced genomic scaffold scf7180000491028, whole genome shotgun sequence
Drosophila erecta	gnl|dere|scaffold_4929 type=golden_path_region; loc=scaffold_4929:1..26641161; ID=scaffold_4929; dbxref=GB:CH954177
Drosophila eugracilis	gi|459206169|gb|KB464972.1| Drosophila eugracilis unplaced genomic scaffold scf7180000409122, whole genome shotgun sequence
Drosophila ficusphila	gi|459201587|gb|KB457400.1| Drosophila ficusphila unplaced genomic scaffold scf7180000453936, whole genome shotgun sequence
Drosophila grimshawi	gnl|dgri|scaffold_15252 type=golden_path_region; loc=scaffold_15252:1..17193109; ID=scaffold_15252; dbxref=GB:CH916368
Drosophila kikkawai	gi|459202993|gb|KB459629.1| Drosophila kikkawai unplaced genomic scaffold scf7180000302408, whole genome shotgun sequence
Drosophila melanogaster	FlyBase_Annotation_IDs:CG10692-PC. Accession number: AAF53163.2
Drosophila miranda	gi|480995217|gb|CM001520.2| Drosophila miranda strain MSH22 chromosome 4, whole genome shotgun sequence
Drosophila mojavensis	gnl|dmoj|scaffold_6500 type=golden_path_region; loc=scaffold_6500:1..32352404; ID=scaffold_6500; dbxref=GB:CH933807
Drosophila persimilis	gnl|dper|scaffold_8 type=golden_path_region; loc=scaffold_8:1..3966273; ID=scaffold_8; dbxref=GB:CH479187
Drosophila pseudoobscura	gnl|dpse|4_group2 type=golden_path_region; loc=4_group2:1..1235759; ID=4_group2; dbxref=GB:CH379059
Drosophila rhopaloa	gi|452191607|gb|KB448653.1| Drosophila rhopaloa unplaced genomic scaffold scf7180000761302, whole genome shotgun sequence
Drosophila sechellia	gnl|dsec|scaffold_16 type=golden_path_region; loc=scaffold_16:1..1878335; ID=scaffold_16; dbxref=GB:CH480831
Drosophila simulans	gnl|dsim|2L type=golden_path_region; loc=2L:1..22036055; ID=2L; dbxref=GB:CM000361
Drosophila suzukii	scaffold1 [22179055 - 22179114] - [22179153 - 22180145]
Drosophila takahashii	gi|459204042|gb|KB461661.1| Drosophila takahashii unplaced genomic scaffold scf7180000415863, whole genome shotgun sequence
Drosophila virilis	nl|dvir|scaffold_12963 type=golden_path_region; loc=scaffold_12963:1..20206255; ID=scaffold_12963; dbxref=GB:CH940649
Drosophila willistoni	Dwil\GK21086-PB type=CDS; loc=scf2_1100000004851:join(40528..40546,40598..41559); name=Dwil\GK21086-RB; dbxref=FlyBase:FBpp0378827 / Garcia et al. 2007
Drosophila yakuba	gnl|dyak|2L type=golden_path_region; loc=2L:1..22324452; ID=2L; dbxref=GB:CM000157
