# Supplementary figures and images for: Evolution of DNMT2 in drosophilids: Evidence for positive and purifying selection and insights into new protein (pathways) interactions
Source: Genet Mol Biol. 2018 Mar 26;41(1 Suppl 1):215–34. doi: 10.1590/1678-4685-GMB-2017-0056 (PMC5913717; doi:10.1590/1678-4685-GMB-2017-0056)

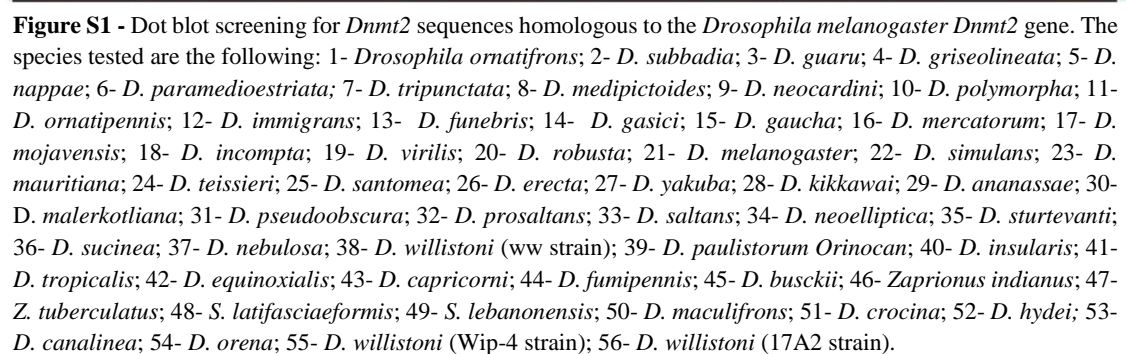

Supplement: Supplementary file 3 [file 1415-4757-GMB-41-01-2017-0056-s007.pdf]
