# Supplementary material for: Evolution of DNMT2 in drosophilids: Evidence for positive and purifying selection and insights into new protein (pathways) interactions
Source: Genet Mol Biol. 2018 Mar 26;41(1 Suppl 1):215–34. doi: 10.1590/1678-4685-GMB-2017-0056 (PMC5913717; doi:10.1590/1678-4685-GMB-2017-0056)
Supplement: Supplementary file 4 [file 1415-4757-GMB-41-01-2017-0056-s008.pdf]

Supplementary Material to "Evolution of DNMT2 in drosophilids: Evidence for positive and purifying selection and insights into new protein (pathways) interactions"

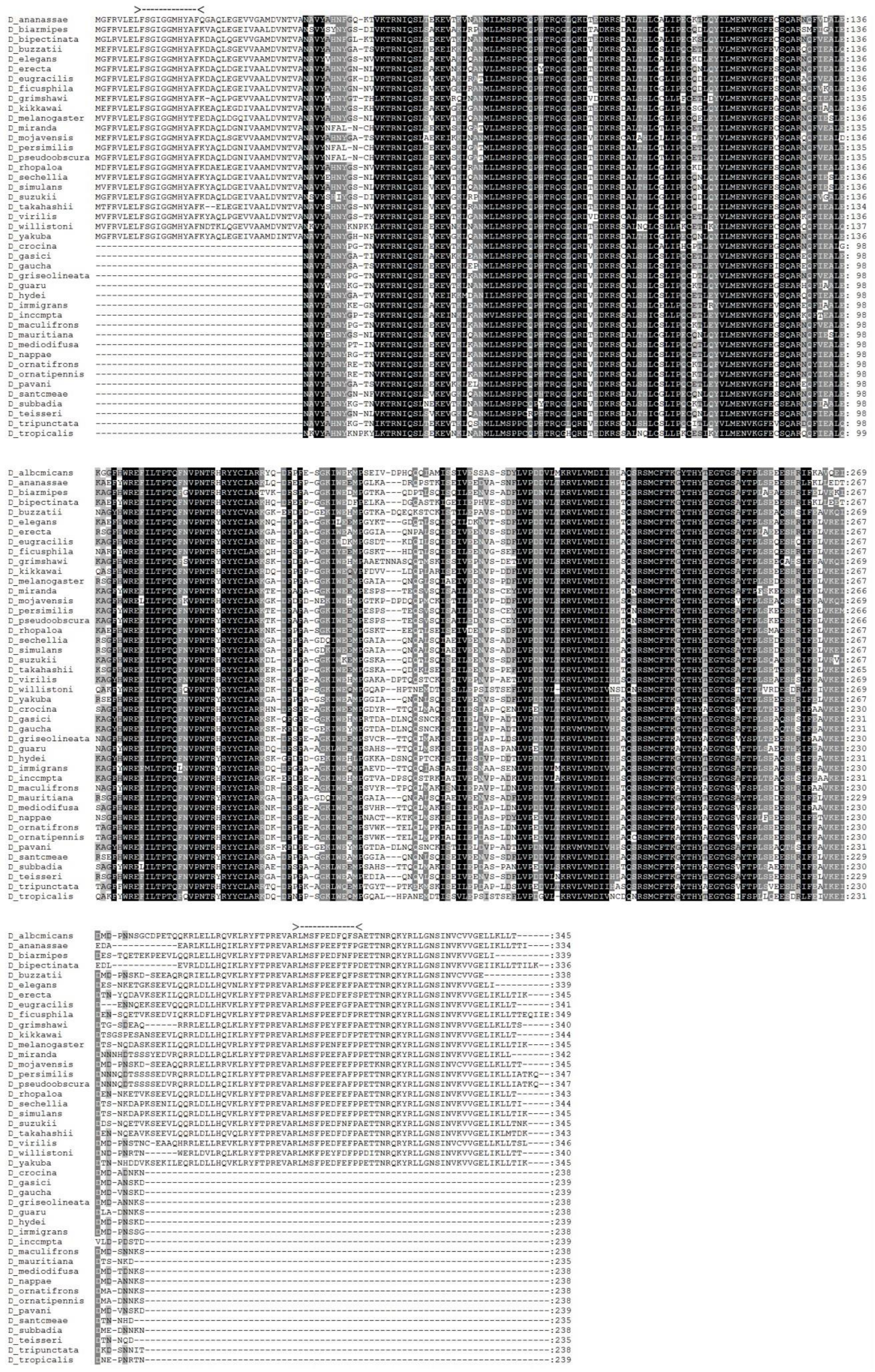

Figure S2 - Multiple sequence alignment of DNMT2 showing the high conservation among *Drosophila* species. Dashes indicate unknown amino acids. Black boxes indicate conserved in all sequences. The positions of PCR primers are indicated by dashed lines.
