# Supplementary material for: Evolution of DNMT2 in drosophilids: Evidence for positive and purifying selection and insights into new protein (pathways) interactions
Source: Genet Mol Biol. 2018 Mar 26;41(1 Suppl 1):215–34. doi: 10.1590/1678-4685-GMB-2017-0056 (PMC5913717; doi:10.1590/1678-4685-GMB-2017-0056)
Supplement: Supplementary file 5 [file 1415-4757-GMB-41-01-2017-0056-s003.pdf]

Supplementary Material to “Evolution of DNMT2 in drosophilids: Evidence for positive and purifying selection and insights into new protein (pathways) interactions”

Table S3 - GenBank accession numbers for nucleotide sequences obtained by DNA sequencing performed by Macrogen Inc.

Accession numbers		
Drosophila tropicalis (clone 1)	Seq1	KJ873136
Drosophila tropicalis (clone 2)	Seq2	KJ873137
Drosophila tripunctata	Seq3	KJ873138
Drosophila teissieri (clone 4)	Seq4	KJ873139
Drosophila teissieri (clone 3)	Seq5	KJ873140
Drosophila teissieri (clone 2)	Seq6	KJ873141
Drosophila subbadia	Seq7	KJ873142
Drosophila pavani	Seq8	KJ873143
Drosophila ornatipennis (clone 1)	Seq9	KJ873144
Drosophila ornatipennis (clone 2)	Seq10	KJ873145
Drosophila ornatifrons (clone 3)	Seq11	KJ873146
Drosophila ornatifrons (clone 2)	Seq12	KJ873147
Drosophila ornatifrons (clone 1)	Seq13	KJ873148
Drosophila nappae	Seq14	KJ873149
Drosophila mediodifusa	Seq15	KJ873150
Drosophila maculifrons	Seq16	KJ873151
Drosophila incompta	Seq17	KJ873152
Drosophila immigrans	Seq18	KJ873153
Drosophila hydei	Seq19	KJ873154
Drosophila guaru	Seq20	KJ873155
Drosophila griseolineata	Seq21	KJ873156
Drosophila gaucha	Seq22	KJ873157
Drosophila gasici	Seq23	KJ873158
Drosophila crocina	Seq24	KJ873159
