# Supplementary material for: Evolution of DNMT2 in drosophilids: Evidence for positive and purifying selection and insights into new protein (pathways) interactions
Source: Genet Mol Biol. 2018 Mar 26;41(1 Suppl 1):215–34. doi: 10.1590/1678-4685-GMB-2017-0056 (PMC5913717; doi:10.1590/1678-4685-GMB-2017-0056)
Supplement: Supplementary file 6 [file 1415-4757-GMB-41-01-2017-0056-s004.pdf]

Supplementary Material to "Evolution of DNMT2 in drosophilids: Evidence for positive and purifying selection and insights into new protein (pathways) interactions"

Table S4 - Standard error estimate(s) from estimates of evolutionary divergence between sequence pairs of different Drosophilidae species groups. Amino acid standard error is given below the diagonal. The nucleotide standard error is provided above the diagonal. The values are given in percentage.

Groups		1	2	3	4	5	6	7	8	9	10	11	12	13
1	calloptera		1.62	1.51	1.11	0.84	1.38	1.31	1.51	1.63	1.39	0.90	1.50	1.63
2	flavopilosa	2.82		1.52	1.53	1.45	1.33	1.31	1.25	1.56	1.14	1.32	1.31	1.55
3	grimshawi	2.79	2.57		1.55	1.43	1.36	1.37	1.40	1.56	1.34	1.34	1.37	1.63
4	guaramunu	1.75	2.64	2.69		0.96	1.29	1.28	1.42	1.60	1.37	0.76	1.40	1.58
5	guarani	1.38	2.73	2.65	1.59		1.22	1.19	1.33	1.44	1.24	0.82	1.32	1.49
6	immigrans	2.31	2.44	2.48	2.23	2.21		1.18	1.34	1.38	1.21	1.09	1.29	1.48
7	melanogaster	2.53	2.57	2.48	2.48	2.44	2.36		1.30	1.11	1.25	1.13	1.21	1.29
8	mesophragmatica	2.62	2.31	2.55	2.42	2.52	2.41	2.52		1.57	1.07	1.23	1.24	1.53
9	obscura	2.84	2.89	2.66	2.73	2.65	2.55	2.18	2.78		1.47	1.38	1.49	1.54
10	repleta	2.54	2.10	2.34	2.33	2.44	2.31	2.44	1.70	2.68		1.18	1.14	1.46
11	tripunctata	1.66	2.57	2.55	1.34	1.55	2.12	2.37	2.33	2.60	2.24		1.20	1.42
12	virilis	2.56	2.13	2.33	2.32	2.46	2.14	2.36	1.92	2.68	1.93	2.24		1.55
13	willistoni	2.94	2.91	2.84	2.87	2.84	2.64	2.66	2.90	2.71	2.75	2.79	2.82	
