# Supplementary material for: Evolution of DNMT2 in drosophilids: Evidence for positive and purifying selection and insights into new protein (pathways) interactions
Source: Genet Mol Biol. 2018 Mar 26;41(1 Suppl 1):215–34. doi: 10.1590/1678-4685-GMB-2017-0056 (PMC5913717; doi:10.1590/1678-4685-GMB-2017-0056)
Supplement: Supplementary file 7 [file 1415-4757-GMB-41-01-2017-0056-s005.pdf]

# Supplementary Material to "Evolution of DNMT2 in drosophilids: Evidence for positive and purifying selection and insights into new protein (pathways) interactions"

**Table S5** - Estimates of Average Percentual Divergence over Sequence Pairs Groups. The values are given in percentage.

| | Within Groups | | | |
|---|---|---|---|---|
| Subgenus | Nucleotides | S.E. | Amino Acids | S.E. |
| *Drosophila* | 21.94 | 0.94 | 17.89 | 1.65 |
| *Sophophora* | 22.65 | 0.89 | 18.17 | 1.62 |
| | Between Groups | | | |
| *Drosophila* vs *Sophophora* | 31.37 | 1.17 | 25.35 | 2.14 |
