# Supplementary material for: Evolution of DNMT2 in drosophilids: Evidence for positive and purifying selection and insights into new protein (pathways) interactions
Source: Genet Mol Biol. 2018 Mar 26;41(1 Suppl 1):215–34. doi: 10.1590/1678-4685-GMB-2017-0056 (PMC5913717; doi:10.1590/1678-4685-GMB-2017-0056)
Supplement: Supplementary file 8 [file 1415-4757-GMB-41-01-2017-0056-s009.pdf]

Supplementary Material to "Evolution of DNMT2 in drosophilids: Evidence for positive and purifying selection and insights into new protein (pathways) interactions"

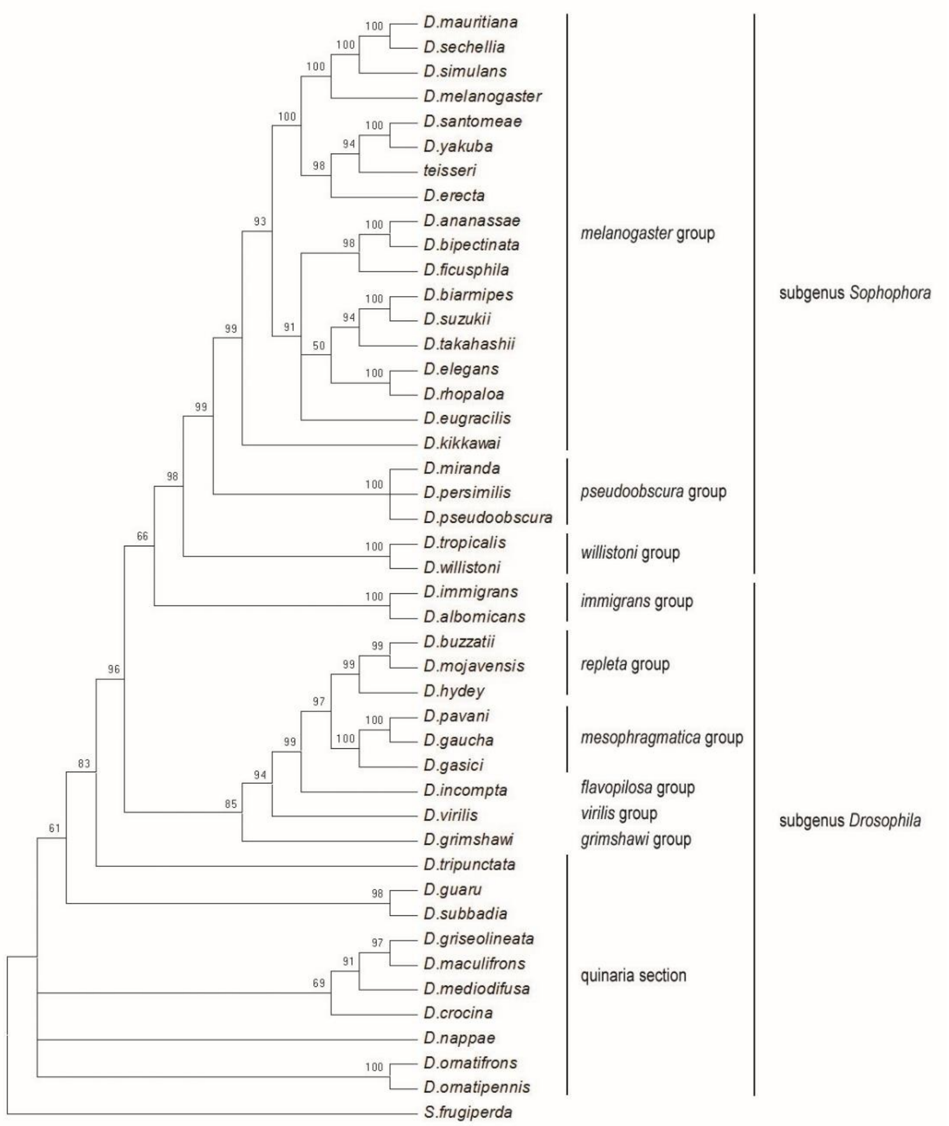

Figure S3 - Bayesian phylogenetic analysis of *Dnmt2* using nucleotide sequences alignment with the GTR + I + G model. Sequence of *Spodoptera frugiperda* was used as outgroup.
