# Supplementary material for: Evolution of DNMT2 in drosophilids: Evidence for positive and purifying selection and insights into new protein (pathways) interactions
Source: Genet Mol Biol. 2018 Mar 26;41(1 Suppl 1):215–34. doi: 10.1590/1678-4685-GMB-2017-0056 (PMC5913717; doi:10.1590/1678-4685-GMB-2017-0056)
Supplement: Supplementary file 9 [file 1415-4757-GMB-41-01-2017-0056-s006.pdf]

Supplementary Material to "Evolution of DNMT2 in drosophilids: Evidence for positive and purifying selection and insights into new protein (pathways) interactions"

Table S6 - ERC values for 36 genes obtained by Group ERC Analysis tool and STRING database. The matrix shows all pairwise ERC values between genes below the diagonal and respectively p-values above the diagonal. Cells are shaded red according to the intensity of their deviation from the null expectation.

	Protein	1	2	3	4	5	6	7	8	9	10	11	12	13	14	15	16	17	18	19	20	21	22	23	24	25	26	27	28	29	30	31	32	33	34	35	36
1	CG10262	N/A	0.0267	0.0870	0.0746	0.3121	0.1894	0.8242	0.1642	0.3975	0.1200	0.3011	0.0671	0.4888	0.8027	0.3595	0.8446	0.9090	0.9445	0.5092	0.9381	0.9016	0.7916	0.8212	0.4777	0.6838	0.8603	0.8008	0.8175	0.9699	0.8959	0.7649	0.9397	0.8650	0.6683	0.5926	0.4963
2	CG6133	0.445	N/A	0.0025	0.2969	0.7554	0.4664	0.9058	0.4664	0.6381	0.1506	0.4506	0.1630	0.2161	0.3430	0.2375	0.4664	0.8818	0.4664	0.8307	0.8778	0.8140	0.3697	0.7751	0.6189	0.6106	0.8216	0.7008	0.7492	0.4664	0.5333	0.7384	0.4664	0.4415	0.2985	0.4463	0.4664
3	Thor	0.330	0.692	N/A	0.0277	0.3575	0.3313	0.9334	0.3011	0.5814	0.3169	0.9111	0.6423	0.7483	0.7049	0.2266	0.6023	0.7473	0.4310	0.6711	0.9063	0.7976	0.1844	0.5842	0.3227	0.6726	0.5916	0.8271	0.9957	0.5799	0.1838	0.8734	0.4031	0.3538	0.0809	0.7808	0.3801
4	CG17124	0.349	0.143	0.681	N/A	0.1266	0.5245	0.7256	0.3273	0.5086	0.5086	0.9194	0.7266	0.9112	0.6945	0.2117	0.5228	0.5673	0.6855	0.7784	0.9621	0.9076	0.5346	0.7417	0.5310	0.6283	0.5892	0.7378	0.7360	0.9349	0.8429	0.9415	0.8528	0.7118	0.3322	0.6526	0.2061
5	Rpd3	0.132	-0.192	0.112	0.430	N/A	0.0707	0.9298	0.9040	0.4322	0.8407	0.9839	0.4571	0.7957	0.9322	0.3414	0.7457	0.7457	0.9281	0.8005	0.9166	0.8908	0.5656	0.5519	0.2268	0.2871	0.4307	0.5873	0.2063	0.7342	0.4400	0.2407	0.6554	0.8760	0.5007	0.7768	0.2836
6	Su(var)3-9	0.226	0.000	0.140	-0.024	0.477	N/A	0.9741	0.9724	0.2433	0.5395	0.4721	0.0499	0.2595	0.5373	0.5268	0.9166	0.7751	0.9575	0.8493	0.8261	0.8428	0.7644	0.7706	0.5386	0.4001	0.6370	0.6259	0.1060	0.6748	0.3435	0.1053	0.2066	0.5886	0.4790	0.4225	0.4097
7	MBD-like	-0.223	-0.349	-0.557	-0.235	-0.494	-0.623	N/A	0.0986	0.7075	0.4001	0.1168	0.5913	0.4760	0.2360	0.5402	0.1662	0.2105	0.0751	0.0681	0.0747	0.1275	0.3487	0.4072	0.6351	0.5382	0.3116	0.4331	0.6999	0.4679	0.8006	0.7666	0.5188	0.3229	0.7778	0.2503	0.6498
8	Eno	0.250	0.000	0.176	0.173	-0.450	-0.616	0.437	N/A	0.4037	0.0928	0.1698	0.4179	0.4147	0.2632	0.1643	0.1637	0.5291	0.1975	0.1913	0.2652	0.2834	0.3237	0.4462	0.6079	0.5660	0.5368	0.6481	0.8749	0.6462	0.7919	0.9030	0.8716	0.5313	0.4443	0.8036	0.8879
9	CG7470	0.074	-0.096	-0.104	-0.011	0.032	0.209	-0.175	0.082	N/A	0.0327	0.1782	0.1222	0.4789	0.4339	0.8101	0.9769	0.9469	0.9469	0.8913	0.9049	0.9669	0.9971	0.8153	0.9778	0.9392	0.9625	0.9292	0.8964	0.9042	0.9114	0.4347	0.6708	0.4741	0.5100	0.1410	0.1265
10	egg	0.290	0.293	0.158	-0.011	-0.355	-0.016	0.089	0.457	0.492	N/A	0.0558	0.0886	0.3421	0.1929	0.2158	0.6974	0.9358	0.7715	0.5351	0.7021	0.8695	0.7856	0.9304	0.9873	0.8982	0.9175	0.6842	0.9220	0.7652	0.7932	0.6224	0.7122	0.3489	0.2205	0.3697	0.2827
11	Su(var)3-3	0.140	0.014	-0.515	-0.521	-0.649	0.011	0.402	0.333	0.231	0.555	N/A	0.0901	0.0832	0.0721	0.4440	0.4666	0.5479	0.5882	0.3433	0.1926	0.3291	0.8081	0.7511	0.9167	0.6510	0.8003	0.5298	0.6624	0.5479	0.7499	0.5440	0.6116	0.4364	0.7527	0.2516	0.6874

Protein	1	2	3	4	5	6	7	8	9	10	11	12	13	14	15	16	17	18	19	20	21	22	23	24	25	26	27	28	29	30	31	32	33	34	35	36
12 Rel	0.360	0.279	-0.166	-0.236	0.010	0.486	-0.066	0.068	0.300	0.479	0.477	N/A	0.0294	0.1886	0.2468	0.8406	0.9550	0.9801	0.6515	0.7448	0.7742	0.8212	0.9855	0.8723	0.4347	0.7816	0.4644	0.4537	0.7830	0.6698	0.2971	0.5333	0.7103	0.7777	0.4427	0.6557
13 CG9973	0.013	0.220	-0.289	-0.502	-0.300	0.194	0.022	0.071	-0.015	0.128	0.493	0.544	N/A	0.0403	0.4346	0.3629	0.6238	0.5536	0.4910	0.1924	0.1836	0.2725	0.7717	0.5432	0.0850	0.5965	0.2313	0.2830	0.3399	0.4020	0.3114	0.5762	0.7275	0.7862	0.7047	0.9503
14 CG6712	-0.203	0.104	-0.236	-0.200	-0.497	-0.014	0.248	0.217	0.010	0.311	0.516	0.258	0.486	N/A	0.0617	0.0259	0.1270	0.1801	0.2324	0.1249	0.1443	0.1664	0.6157	0.6674	0.1078	0.1859	0.0539	0.1397	0.2696	0.4426	0.5660	0.2815	0.0795	0.4338	0.3285	0.6756
15 Su(var)205	0.100	0.200	0.274	0.309	0.118	-0.005	-0.021	0.339	-0.279	0.281	0.041	0.193	0.039	0.466	N/A	0.0340	0.4751	0.4864	0.2740	0.4232	0.4516	0.0794	0.6066	0.4189	0.1395	0.2660	0.1736	0.2337	0.3626	0.3831	0.5942	0.6658	0.4725	0.2569	0.9258	0.7505
16 mei-S332	-0.242	0.000	-0.125	-0.023	-0.244	-0.463	0.328	0.340	-0.547	-0.204	0.019	-0.283	0.093	0.564	0.602	N/A	0.0253	0.0284	0.1485	0.0297	0.0180	0.0047	0.1072	0.0903	0.0312	0.0248	0.4685	0.0943	0.0643	0.1754	0.5163	0.3803	0.1969	0.4272	0.8071	0.8963
17 CG14618	-0.315	-0.318	-0.288	-0.067	-0.244	-0.251	0.274	-0.027	-0.463	-0.526	-0.052	-0.424	-0.088	0.354	0.014	0.662	N/A	0.0093	0.0398	0.0541	0.0117	0.0249	0.1015	0.1111	0.1780	0.0123	0.0732	0.0489	0.0775	0.1203	0.4843	0.1507	0.0889	0.4458	0.3271	0.6393
18 CG14906	-0.368	0.000	0.037	-0.190	-0.490	-0.565	0.479	0.298	-0.463	-0.285	-0.086	-0.492	-0.036	0.285	0.003	0.650	0.731	N/A	0.0089	0.0125	0.0025	0.0008	0.0459	0.0879	0.2154	0.0266	0.1378	0.1770	0.0431	0.1251	0.4492	0.1084	0.0538	0.2508	0.5496	0.8672
19 Atac3	0.000	-0.263	-0.199	-0.299	-0.305	-0.343	0.495	0.304	-0.370	-0.051	0.136	-0.124	0.000	0.226	0.216	0.386	0.593	0.782	N/A	0.0032	0.0070	0.0057	0.0492	0.1193	0.3912	0.0180	0.0818	0.2100	0.1437	0.1325	0.3912	0.3912	0.0416	0.2551	0.3912	0.8704
20 CTCF	-0.355	-0.313	-0.505	-0.635	-0.470	-0.312	0.480	0.215	-0.388	-0.209	0.313	-0.195	0.250	0.357	0.065	0.643	0.555	0.758	0.801	N/A	0.0004	0.0032	0.0238	0.1094	0.1260	0.0641	0.1074	0.1074	0.0153	0.1087	0.1688	0.1578	0.1556	0.5051	0.6241	0.9606
21 CG16863	-0.307	-0.249	-0.349	-0.495	-0.429	-0.333	0.384	0.196	-0.512	-0.404	0.151	-0.220	0.258	0.328	0.036	0.698	0.713	0.844	0.763	0.932	N/A	0.0009	0.0262	0.0448	0.0905	0.0267	0.0693	0.0804	0.0165	0.0794	0.2525	0.1433	0.1591	0.4930	0.6628	0.9689
22 sna	-0.193	0.084	0.334	-0.034	-0.070	-0.237	0.134	0.160	-0.685	-0.300	-0.298	-0.263	0.170	0.300	0.483	0.796	0.650	0.874	0.773	0.833	0.912	N/A	0.1107	0.0146	0.0721	0.0278	0.0655	0.0518	0.0127	0.0411	0.3520	0.2044	0.1772	0.1765	0.8855	0.8899
23 homer	-0.220	-0.211	-0.106	-0.253	-0.058	-0.245	0.083	0.044	-0.284	-0.512	-0.237	-0.516	-0.205	-0.088	-0.096	0.457	0.457	0.622	0.587	0.708	0.713	0.524	N/A	0.0026	0.0788	0.0196	0.1536	0.1112	0.0397	0.0661	0.0903	0.1573	0.1939	0.4617	0.7484	0.9587

Protein	1	2	3	4	5	6	7	8	9	10	11	12	13	14	15	16	17	18	19	20	21	22	23	24	25	26	27	28	29	30	31	32	33	34	35	36
**24** Rpp20	0.021	-0.080	0.150	-0.030	0.234	-0.015	-0.107	-0.095	-0.551	-0.698	-0.449	-0.316	-0.028	-0.129	0.070	0.489	0.441	0.513	0.440	0.506	0.651	0.797	0.777	N/A	0.0127	0.0186	0.1018	0.0495	0.0703	0.0950	0.1915	0.2152	0.3997	0.5476	0.8641	0.9423
**25** RhoGEF4	-0.105	-0.073	-0.200	-0.128	0.167	0.072	-0.019	-0.058	-0.445	-0.447	-0.140	0.022	0.393	0.384	0.379	0.638	0.337	0.313	0.000	0.480	0.541	0.602	0.498	0.716	N/A	0.0322	0.0241	0.0250	0.1488	0.1788	0.1462	0.3951	0.5526	0.7507	0.8909	0.9738
**26** Dnmt2	-0.259	-0.255	-0.114	-0.088	0.033	-0.103	0.171	-0.034	-0.499	-0.484	-0.290	-0.226	-0.067	0.278	0.224	0.663	0.711	0.693	0.699	0.589	0.712	0.741	0.650	0.680	0.577	N/A	0.0179	0.0049	0.0392	0.0339	0.1800	0.0283	0.0435	0.3357	0.5663	0.8596
**27** Orc2	-0.201	-0.145	-0.384	-0.249	-0.089	-0.090	0.060	-0.133	-0.430	-0.190	-0.036	0.000	0.210	0.485	0.333	0.000	0.512	0.425	0.509	0.508	0.585	0.616	0.375	0.470	0.609	0.686	N/A	0.0044	0.4939	0.0428	0.1165	0.4939	0.1024	0.3770	0.6674	0.6884
**28** hay	-0.216	-0.186	-0.794	-0.247	0.261	0.372	-0.168	-0.384	-0.378	-0.492	-0.151	0.007	0.159	0.334	0.262	0.481	0.572	0.369	0.297	0.508	0.563	0.653	0.440	0.582	0.605	0.771	0.741	N/A	0.0082	0.0028	0.0493	0.0548	0.1092	0.2091	0.6427	0.4932
**29** hop	-0.420	0.000	-0.102	-0.557	-0.231	-0.140	0.028	-0.131	-0.387	-0.278	-0.052	-0.228	0.110	0.186	0.126	0.540	0.503	0.629	0.399	0.747	0.762	0.805	0.588	0.528	0.351	0.602	0.000	0.774	N/A	0.0033	0.0940	0.0723	0.1083	0.2030	0.6938	0.8344
**30** mms4	-0.300	-0.009	0.336	-0.387	0.025	0.120	-0.275	-0.280	-0.400	-0.309	-0.236	-0.138	0.061	0.039	0.106	0.344	0.426	0.445	0.418	0.507	0.565	0.686	0.521	0.483	0.313	0.622	0.542	0.831	0.845	N/A	0.0273	0.0506	0.1272	0.1237	0.6868	0.8179
**31** mus209	-0.168	-0.176	-0.450	-0.578	0.218	0.373	-0.233	-0.432	0.009	-0.134	-0.049	0.143	0.136	-0.051	-0.085	-0.010	0.020	0.052	0.000	0.409	0.295	0.185	0.479	0.336	0.355	0.349	0.397	0.621	0.551	0.703	N/A	0.0923	0.2352	0.2943	0.5754	0.7986
**32** pnt	-0.359	0.000	0.064	-0.402	-0.153	0.247	-0.002	-0.380	-0.157	-0.220	-0.105	-0.034	-0.052	0.176	-0.151	0.087	0.375	0.473	0.000	0.426	0.447	0.377	0.370	0.307	0.097	0.644	0.000	0.609	0.596	0.631	0.476	N/A	0.0010	0.1602	0.1829	0.6397
**33** Su(var)2-10	-0.264	0.023	0.116	-0.220	-0.402	-0.057	0.160	-0.029	-0.011	0.122	0.050	-0.169	-0.167	0.434	0.016	0.311	0.480	0.597	0.608	0.429	0.424	0.421	0.323	0.109	-0.025	0.590	0.421	0.497	0.524	0.481	0.264	0.811	N/A	0.0680	0.1069	0.4294
**34** Cap	-0.095	0.142	0.512	0.168	-0.016	0.004	-0.246	0.045	-0.040	0.274	-0.239	-0.223	-0.217	0.047	0.235	0.041	0.056	0.267	0.239	0.002	0.011	0.422	0.031	-0.031	-0.209	0.172	0.108	0.343	0.359	0.488	0.193	0.352	0.461	N/A	0.7464	0.5053

	Protein	1	2	3	4	5	6	7	8	9	10	11	12	13	14	15	16	17	18	19	20	21	22	23	24	25	26	27	28	29	30	31	32	33	34	35	36
**35**	CG13035	-0.043	0.018	-0.328	-0.153	-0.279	0.053	0.233	-0.293	0.274	0.101	0.237	0.016	-0.148	0.136	-0.490	-0.278	0.170	-0.037	0.000	-0.122	-0.155	-0.506	-0.232	-0.383	-0.381	-0.043	-0.109	-0.126	-0.206	-0.197	-0.072	0.321	0.399	-0.228	N/A	0.0225
**36**	Hsp22	0.008	0.000	0.089	0.316	0.171	0.064	-0.120	-0.406	0.293	0.195	-0.175	-0.128	-0.427	-0.135	-0.235	-0.385	-0.110	-0.408	-0.381	-0.625	-0.634	-0.515	-0.557	-0.533	-0.567	-0.364	-0.130	0.000	-0.398	-0.357	-0.300	-0.127	0.057	-0.008	0.552	N/A
